# Microendemicity in the northern Hajar Mountains of Oman and the United Arab Emirates with the description of two new species of geckos of the genus *Asaccus* (Squamata: Phyllodactylidae)

**DOI:** 10.7717/peerj.2371

**Published:** 2016-08-18

**Authors:** Salvador Carranza, Marc Simó-Riudalbas, Sithum Jayasinghe, Thomas Wilms, Johannes Els

**Affiliations:** 1 Animal Biodiversity and Evolution, Institute of Evolutionary Biology (CSIC-Pompeu Fabra University), Barcelona, Spain; 2 Herpetology and Freshwater Fishes, Breeding Centre for Endangered Arabian Wildlife, Environment and Protected Areas Authority, Al Sharjah, United Arab Emirates; 3 Allwetterzoo Münster, Münster, Germany

**Keywords:** Phylogeny, Systematics, Arabia, Diversification, Reptiles, Speciation, Taxonomy, Evolution, Mountains, Biogeography

## Abstract

**Background:**

The Hajar Mountains of Oman and the United Arab Emirates (UAE) is the highest mountain range in Eastern Arabia. As a result of their old geological origin, geographical isolation, complex topography and local climate, these mountains provide an important refuge for endemic and relict species of plants and animals with strong Indo-Iranian affinities. Among vertebrates, the rock climbing nocturnal geckos of the genus *Asaccus* represent the genus with the highest number of endemic species in the Hajar Mountains. Recent taxonomic studies on the Zagros populations of *Asaccus* have shown that this genus is much richer than it was previously thought and preliminary morphological and molecular data suggest that its diversity in Arabia may also be underestimated.

**Methods:**

A total of 83 specimens originally classified as *Asaccus caudivolvulus* (including specimens of the two new species described herein), six other *Asaccus* species from the Hajar and the Zagros Mountains and two representatives of the genus *Haemodracon* were sequenced for up to 2,311 base pairs including the mitochondrial *12S* and *cytb* and the nuclear *c-mos*, *MC1R* and *ACM4* genes. Phylogenetic relationships were inferred using both Bayesian and maximum-likelihood approaches and the former method was also used to calibrate the phylogenetic tree. Haplotype networks and phylogenetic trees were inferred from the phased nuclear genes only. Sixty-one alcohol-preserved adult specimens originally classified as *Asaccus caudivolvulus* from the northern Hajar Mountains were examined for 13 morphometric and the five meristic variables using multivariate methods and were also used to diagnose and describe the two new species.

**Results:**

The results of the molecular and morphological analyses indicate that the species originally classified as *Asaccus caudivolvulus* is, in fact, an assemblage of three different species that started diversifying during the Mid-Miocene. The molecular phylogenies consistently recovered the Hajar endemic *A. montanus* as sister taxon to all the other *Asaccus* species included in the analyses, rendering the Arabian species of *Asaccus* polyphyletic.

**Discussion:**

Using this integrative approach we have uncovered a very old diversification event that has resulted in a case of microendemicity, where three morphologically and ecologically similar medium-sized lizard species coexist in a very short and narrow mountain stretch. *Asaccus caudivolvulus* is restricted to a small coastal area of the UAE and at risk from heavy development, while the two new species described herein are widely distributed across the northern tip of the Hajar Mountains and seem to segregate in altitude when found in close proximity in the Musandam Peninsula (Oman). Similarly to other integrative analyses of Hajar reptiles, this study highlights the high level of diversity and endemicity of this arid mountain range, underscoring its status as one of the top hotspots of reptile diversity in Arabia.

## Introduction

**Figure 1:**
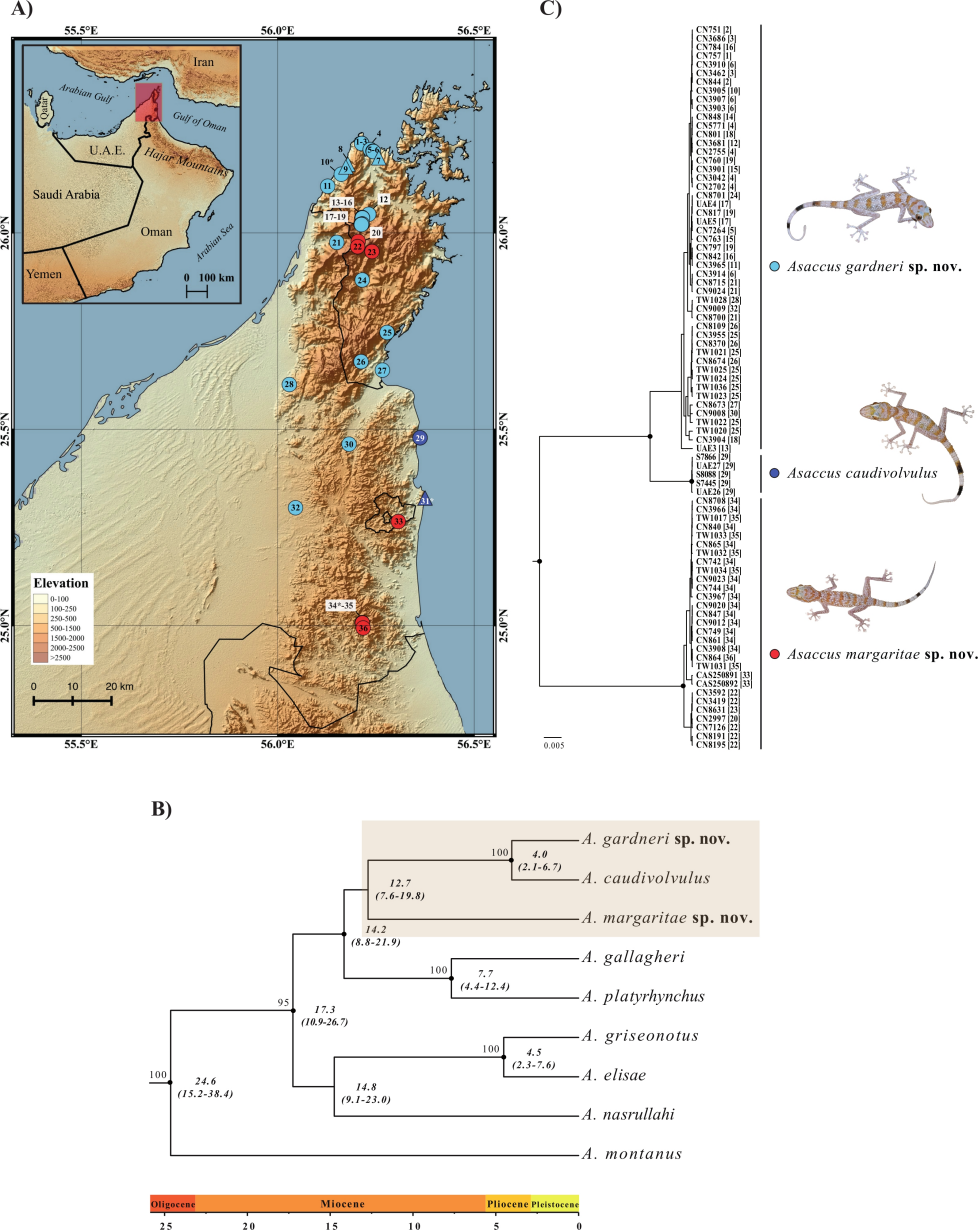
Geographical distribution and phylogenetic relationships. (A) Map of the northern Hajar Mountains showing the localities of examined material. Triangles represent specimens used in the morphological analysis only (without genetic data) whereas circles indicate samples included in the phylogenetic analyses (most of them also used in the morphological analysis); see Table S1. Type localities of the three species are labeled with an asterisk (*). (B) Bayesian inference tree of 9 *Asaccus* species based on the concatenated sequences of two mitochondrial (*12S* and *cytb*) and three nuclear (*c-mos*, *MC1R* and *ACM4*) genes. Black dots indicate posterior probability values ≥0.95 in the Bayesian analysis and bootstrap values ≥70% in the maximum-likelihood analysis (see Fig. S1) are shown next to the nodes. Age estimates are in italics below the nodes and include the mean and the HPD 95% confidence interval in brackets. The tree was rooted using one specimen of *Haemodracon riebeckii* and one specimen of *H. trachyrhinus* (not included in the figure). (C) Bayesian inference tree of 83 *Asaccus* based on the same concatenated genes. Black dots indicate posterior probability values ≥0.95. Each sequence labeled with the specimen code followed by the locality code in square brackets (see Fig. 1A). Detailed information on the samples included in both phylogenetic trees is given in Table S1.

The Hajar Mountain range is the highest in eastern Arabia, forming a spectacular isolated wall of mountains that rises dramatically from the ocean below. It runs northwest to southeast in a 650 km arc paralleling the Oman and United Arab Emirates (UAE) coast of the Gulf of Oman and is surrounded by the sea to the east and by a very large desert to the west (Edgell, 2006; see Fig. 1A). Despite being close to the sea and the only area in eastern Arabia with habitats above 2,000 m in elevation, rainfall is low (with average precipitation estimates of 300 mm over much of its range), evapotranspiration is high and the almost treeless, barren nature of the terrain classifies it as a mountain desert (Mandaville, 1977; Edgell, 2006). This contrasts sharply with the other two main mountain ranges in Arabia, the Western Mountains of Yemen and Saudi Arabia and the Dhofar Mountains of southern Oman and eastern Yemen. Both these ranges are partially or totally affected by the moisture laden southwest monsoon winds that blow against the sea-facing cliffs between July and August and that are responsible for the unique green vegetation on the southward (sea) side of these mountain ranges (Sale, 1980).

The Hajar Mountains have a complex geological history and have long been known to have more structural and petrological features in common with the Zagros Mountains of southwestern Iran than with neighboring parts of Arabia (Mandaville, 1977). The mountain core is of Mesozoic sediments, in part metamorphosed, uplifted and folded during the Oligocene and Miocene by the effect of the opening of the Gulf of Aden and a series of plate tectonics that affected Oman between 4–6 Ma (Bosworth, Huchon & McClay, 2005; Glennie, 2006). As a result of its old geological origin, geographical isolation, complex topography with many high peaks and very deep canyons (wadis) that cut through the mountains and a local microclimate, the Hajar Mountains have a relatively rich fauna and flora, providing an important refuge for endemic and relict species mostly of Indo-Iranian origin (Mandaville, 1977 and references therein). With 17 species almost exclusively restricted to this mountain region, reptiles are the vertebrate group with the highest level of endemicity and one of the main inhabitants of the Hajar Mountains (Arnold, 1972; Arnold, 1986; Arnold & Gallagher, 1977; Arnold & Gardner, 1994; Carranza & Arnold, 2012; Gardner, 2013; Metallinou et al., 2015; De Pous et al., 2016; Tamar et al., 2016). Of all the endemic reptiles, the geckos of the genus *Asaccus* Dixon & Anderson, 1973 are the ones with the highest level of endemicity (four endemic species) and a group that clearly exemplifies the affinities between the Hajar Mountains in Arabia and the Zagros Mountains of southwest Asia (Torki et al., 2011a; Uetz, Goll & Hallerman, 2016).

The genus *Asaccus* Dixon & Anderson, 1973 was created for Middle Eastern geckos previously placed in the genus *Phyllodactylus* Gray, 1828. The genus *Asaccus* includes small to medium size (snout vent length 40 mm–71 mm), slender, nocturnal, rock climbing geckos with paired terminal scansors in the digits that lack lamellae, without femoral or preanal pores, cloacal sacs or bones and without the left oviduct (only the right oviduct is present and therefore only one egg is laid at a time). For nearly 20 years the genus *Asaccus* comprised just three species; two from the Zagros and one from the Hajar Mountains. However, a new endemic species was described from the Hajar Mountains (*A. montanus* Gardner, 1994) and a taxonomic revision carried out by Arnold & Gardner (1994) led to the description and inclusion of two more Hajar endemics, thereby elevating the number of Arabian species to four. Based on this finding and a morphological phylogeny, the authors hypothesized that *Asaccus* originated in the Hajar Mountains of Arabia and that *A. montanus* was the result of a back colonization from Iran. As a result of a growing scientific interest in the populations of *Asaccus* from the Zagros Mountains, a total of 10 new species endemic to this massif have been described since 1994, increasing the total number of species of *Asaccus* to 16 (four endemic to the Hajar Mountains and 12 to the Zagros Mountains) (Werner, 1895; Arnold, 1972; Dixon & Anderson, 1973; Arnold & Gardner, 1994; Gardner, 1994; Rastegar-Pouyani, 1996; Rastegar-Pouyani, Nilson & Faizi, 2006; Werner, 2006; Afrasiab & Mohamad, 2009; Torki, 2010; Torki et al., 2011a; Torki et al., 2011b). These results suggest that the diversity of *Asaccus* is probably higher in the Zagros than in the Hajar Mountains. Consequently, Rastegar-Pouyani, Nilson & Faizi (2006) proposed the Zagros Mountains as the center of origin of *Asaccus*; a hypothesis that was recently challenged by a partial molecular phylogeny of the genus, in which the Hajar endemic *A. montanus* was recovered as a sister taxon to all the other *Asaccus* species included in the analyses (Papenfuss et al., 2010). Moreover, preliminary morphological (Arnold & Gardner, 1994) and molecular (Papenfuss et al., 2010) data indicate that the diversity of *Asaccus caudivolvulus* Arnold & Gardner, 1994 and *Asaccus gallagheri* Arnold, 1972 in the Hajar Mountains is probably underestimated, something that has been already shown for several other reptile groups such as *Hemidactylus* (Carranza & Arnold, 2012), *Pristurus* (Badiane et al., 2014); *Ptyodactylus* (Metallinou et al., 2015) and *Trachydactylus* (De Pous et al., 2016).

*Asaccus caudivolvulus* was described by Arnold & Gardner (1994) based on collections from two different localities: the type locality in Khor Fakkan, UAE (locality 31 in Fig. 1A) and Khasab, a locality 100 km to the north, in the Musandam Peninsula, Oman (locality 7 in Fig. 1A). In the original description, Arnold & Gardner (1994) observed that the specimens from Khasab and Khor Fakkan differed in some characteristics, most notably the absence of tubercles on the upper arm in the specimens from Khasab. Nevertheless, given the lack of material from intermediate localities, the specimens from Khasab were not described as a new species.

In this manuscript, we describe the findings of a comprehensive sampling effort in the northern Hajar Mountains with the objective of clarifying the systematics of *Asaccus caudivolvulus*. In contrast to the findings of Arnold & Gardner (1994), our morphological and molecular analyses indicate the presence of three different species of medium to large size *Asaccus* within what was previously considered *A. caudivolvulus*. Two of the species are widely distributed across the northern Hajar Mountains of Oman and the UAE and are described herein, while the third species, *A. caudivolvulus*, is endemic to the UAE and its current distribution seems restricted to a short coastal stretch of rocky escarpments placing it at risk from heavy development.

## Material and Methods

### Molecular analyses

#### DNA extraction, amplification and sequence analysis

A total of 89 individuals of *Asaccus* and two *Haemodracon* were included in the molecular study. A list of all specimens with their taxonomic identifications, sample codes, voucher references, geographical distribution data and GenBank accession numbers for all sequenced genes is presented in Table S1. Total genomic DNA was isolated from ethanol-preserved tissue samples using the SpeedTools Tissue DNA Extraction kit (Biotools, Madrid, Spain) following the manufacturer’s protocol. All specimens were sequenced for both strands for two mitochondrial gene fragments: the ribosomal 12S rDNA (*12S*) and the cytochrome b (*cytb*) and three nuclear gene fragments: the oocyte maturation factor MOS (*c-mos*), the melanocortin 1 receptor (*MC1R*) and the acetylcholine receptor M4 (*ACM4*). Primers and PCR conditions for the amplification of all fragments are the same as listed in Table S2 of Metallinou et al. (2015).

Geneious Pro v. 8.0.3 (Biomatters Ltd.) was used for assembling and editing the chromatographs manually. All coding gene fragments started by the first codon and were translated into amino acids to validate the correct reading frame. For the nuclear coding gene fragments, heterozygous positions were identified and coded in both alleles according to IUPAC ambiguity codes. Multiple sequence alignments were performed with the online application of MAFFT v.7 (Katoh & Toh, 2008) applying default parameters (Auto strategy, Gap opening penalty: 1.53, Offset value: 0.0). For the *12S* ribosomal fragment the Q-INS-i strategy was applied, in which the secondary structure of the RNA is considered. SEQPHASE (Flot, 2010) was used to convert the input files and the software PHASE v. 2.1.1 to resolve phased haplotypes (Stephens, Smith & Donnelly, 2001). Default settings of PHASE were used except for phase probabilities that were set as ≥0.7 (see Harrigan, Mazza & Sorenson, 2008). Phased sequences of the nuclear genes were used for the network analyses and also to infer a phylogenetic tree of alleles for each nuclear marker. Inter and intra-specific uncorrected *p*-distances with pairwise deletion were estimated for both mitochondrial gene fragments independently using MEGA v.6 (Tamura et al., 2011).

#### Phylogenetic and network analyses

Two datasets were used for the phylogenetic analyses. Dataset 1 was assembled with the aim of studying the phylogenetic relationships and the divergence times among the different species of *Asaccus* included in this study. The dataset included one representative from *A. caudivolvulus*, one from each of the two new species described herein and six other species of *Asaccus*, three from the Hajar Mountains of Arabia (*A. gallagheri*, *A. montanus* and *A. plathyrhynchus* Arnold & Gardner, 1994) and three from the Zagros Mountains (*A. griseonotus* Dixon & Anderson, 1973, *A. elisae* (Werner, 1895) and *A. nasrullahi* Werner, 2006). Moreover, two specimens of the two Socotran endemic species, *Haemodracon riebeckii* and *H. trachyrhinus*, were used as outgroups based on published evidence (Gamble et al., 2008; Gamble et al., 2011; Gamble et al., 2012; Garcia-Porta et al., 2016a; Garcia-Porta et al., 2016b). This dataset included all described species of *Asaccus* from the Hajar Mountains but was missing nine species from the Zagros Mountains described only based on morphological data: *A. andersoni* Torki et al., 2011b, *A. barani* Torki et al., 2011a, *A. granularis* Torki, 2010, *A. iranicus* Torki et al., 2011a, *A. kermanshanensis* Rastegar-Pouyani, 1996, *A. kurdistanensis* Rastegar-Pouyani, Nilson & Faizi, 2006, *A. saffinae* Afrasiab & Mohamad, 2009, *A. tangestanensis* Torki et al., 2011a and *A. zagrosicus* Torki et al., 2011a. Dataset 2 was assembled to study in detail the phylogeographic relationships of *Asaccus caudivolvulus* and the two new species described herein. This dataset consisted of 83 specimens collected from 36 localities distributed across the north of the Hajar Mountains and the Musandam Peninsula (five *Asaccus caudivolvulus*, 49 specimens of the larger of the two new species described herein and 29 specimens of the smaller of the two new species described herein).

Dataset 1 was analysed with maximum likelihood (ML) and Bayesian inference (BI) methods, whereas dataset 2 was only analysed with Bayesian inference, as it only included ingroup sequences (the three species previously classified as *A. caudivolvulus*). The best-fit partitioning scheme and models of molecular evolution for datasets 1 and 2 were selected with PartitionFinder v.1.1.1 (Lanfear et al., 2012) with the following settings: branch lengths linked, only models available in BEAST evaluated, initial partitions by gene, BIC model selection criterion applied and all partition schemes analysed. The partition scheme and models of sequence evolution selected were *12S* + *cytb*, GTR + G; *c-mos* + *ACM4*, HKY + G and *MC1R*, HKY + G for dataset 1 and *12S* + *cytb*, GTR + I; *c-mos* + *ACM4* + *MC1R*, HKY + I + G dataset 2.

ML analyses of dataset 1 were performed in RAxML v.7.4.2 (Stamatakis, 2006) as implemented in raxmlGUI (Silvestro & Michalak, 2010) with 100 tree searches, using the GTR+G model of sequence evolution and independent model parameters for the three partitions (see above). Reliability of the ML tree was assessed by bootstrap analysis (Felsenstein, 1985) including 1,000 replicates. The software BEAST v.1.8.0 (Drummond et al., 2012) was used for BI and dating analyses. Two individual runs of 5 × 10^7^ generations were carried out for datasets 1 and 2, sampling at intervals of 10,000 generations. The following models and prior specifications were applied, otherwise by default: models of sequence evolution for the different partitions as selected by PartitionFinder (see above); Speciation Yule (dataset 1) and Coalescent Constant Size (dataset 2) tree prior; uncorrelated lognormal clock for mitochondrial genes and strict clock for nuclear ones; random starting tree; base substitution prior Uniform (0,100); alpha prior Uniform (0,10). Substitution and clock models were unlinked and the xml file was manually modified to set Ambiguities=“true” for the nuclear gene partitions in order to account for variability in the heterozygous positions, instead of treating them as missing data. Posterior trace plots and effective sample sizes (ESS) of the runs were monitored in Tracer v1.5 (Rambaut & Drummond, 2013) to ensure convergence. The results of the individual runs were combined in LogCombiner discarding 10% of the samples and the ultrametric tree was produced with TreeAnnotator (both provided with the BEAST package).

Absolute divergence times were estimated from dataset 1 using BEAST with models and prior specifications as above and applying previously calculated mean rates of molecular evolution for the two mitochondrial markers *12S* (mean: 0.00755, stdev: 0.00247) and *cytb* (mean: 0.0228, stdev: 0.00806) (Carranza & Arnold, 2012). Despite the problems associated with using evolutionary rates from other organisms for time tree calibration, the rates inferred by Carranza & Arnold (2012) and applied here correspond with the rates obtained in other independent studies that used different calibration points and different taxa (Metallinou et al., 2012; Sindaco et al., 2012). Indeed, the rates by Carranza & Arnold (2012) have been applied to several different studies for which reliable internal calibration points based on biogeographic events or fossil evidence do not exist (Gómez-Díaz et al., 2012; Hawlitschek & Glaw, 2013; Metallinou & Carranza, 2013; Milá et al., 2013; Šmíd et al., 2013; Vasconcelos & Carranza, 2014; Metallinou et al., 2015; De Pous et al., 2016; Tamar et al., 2016). Tree nodes were considered strongly supported if they received ML bootstrap values ≥70% and posterior probability (pp) support values ≥0.95 (Wilcox et al., 2002; Huelsenbeck & Rannala, 2004).

With the aim of exploring patterns of intra-specific diversity and nuclear allele sharing between the three species previously classified as *Asaccus caudivolvulus*, statistical parsimony networks on the three individual nuclear genes were constructed with the program TCS v.1.21 (Clement, Posada & Crandall, 2000) using default settings (connection limit of 95%) and including only full length sequences.

### Morphological analyses

#### Morphological samples, variables and museums acronyms

A total of 61 alcohol-preserved adult specimens of *Asaccus* from the northern Hajars region of UAE and Oman were examined and included in the morphological analyses. Sixteen voucher specimens were obtained from the Natural History Museum, London, UK (BMNH and SQU), two from the Oman Natural History Museum (ONHM) and 45 from S. Carranza’s field series housed at the Institute of Evolutionary Biology (IBE), Barcelona, Spain (Table S2). Variables for the morphological analyses were selected based on previous taxonomic studies of *Asaccus* (Arnold, 1972; Dixon & Anderson, 1973; Arnold & Gardner, 1994; Gardner, 1994; Rastegar-Pouyani, 1996; Rastegar-Pouyani, Nilson & Faizi, 2006; Werner, 2006; Afrasiab & Mohamad, 2009; Torki, 2010; Torki et al., 2011a; Torki et al., 2011b). The following measurements were taken on the right side of each specimen using a digital caliper with accuracy to the nearest 0.1 mm and were expressed in millimeters: snout-vent length (SVL), distance from tip of the snout to cloaca; trunk length (TrL), distance between the fore and hind limb insertion points; head length (HL), taken axially from tip of the snout to the anterior ear border; head height (HH), taken laterally at anterior ear border; head width (HW), taken at anterior ear border; snout length (SL), from snout to the anterior eye border; snout width (SW), taken dorsally at anterior eye border; eye diameter (ED), maximal longitudinal length of the eye; ear vertical diameter (EVD), maximal transversal length of the ear; humerus length (LHu), from elbow to the insertion of the fore limb on the anterior part of body; ulna length (LUn), from wrist to elbow; femur length (LFe), from knee to the insertion of the hind limb on the posterior side of body; tibia length (LTb), from ankle to knee. Tail length was not measured because many individuals had an unequal regenerated tail or had lost it. In addition to these morphometric variables, five pholidotic (meristic) and one categorical character were collected using a dissecting microscope. Pholidotic characters: number of dorsal tubercles rows (Trow); number of upper labial scales (ULS); number of lower labial scales (LLS); number of expanded lamellae rows under the 4th toe (LT4); number of enlarged scales, distally, under the 4th toe (ST4). Categorical character: tubercles on upper arm (TUA)—1: present, 0: absent.

#### Multivariate analyses

Statistical analyses were used to investigate differences in size and shape between *Asaccus caudivolvulus* and the two new species described herein. The 13 morphometric and five meristic variables were analysed independently and the single categorical character was directly used in the description of the new species (see taxonomic account). All measurements were log_10_-transformed to obtain data normality and increase the homogeneity of variance. The final dataset included 61 specimens, 43 of which (26 males and 17 females) corresponded to the larger of the two new species described, 13 (10 males and three females) to the smaller of the two new species described and only five (four males and one female) to *A. caudivolvulus* (Table 1 and Table S2).

**Table 1:** Descriptive statistics for all variables examined for males and females of *A. gardneri* sp. nov., *A. caudivolvulus* and *A. margaritae* sp. nov. Mean ± Standard Deviation (SD) and range (Min–Max) are given. Abbreviations of characters as explained in the material and methods and in Table S2.

Variable	*A. gardneri***sp. nov.**		*A. caudivolvulus*		*A. margaritae***sp. nov.**	
	Males (*n* = 17)	Females (*n* = 26)	Males (*n* = 4)	Females (*n* = 1)	Males (*n* = 10)	Females (*n* = 3)
	Mean ± SD (Min–Max)	Mean ± SD (Min–Max)	Mean ± SD (Min–Max)	Mean ± SD (Min–Max)	Mean ± SD (Min–Max)	Mean ± SD (Min–Max)
SVL	64.2 ± 4.2 (56.1–70.7)	61 ± 4.7 (51.8–70.7)	60.3 ± 4.5 (53.6–63.2)	58.5	54.7 ± 2 (51.7–58.6)	50.7 ± 5.6 (44.3–54.7)
TrL	26.7 ± 2 (22.4–29.8)	24.8 ± 2.9 (19.8–30)	25.5 ± 2.5 (21.9–27.7)	23.9	22.8 ± 1.9 (18.9–26.1)	20.3 ± 2.7 (17.5–22.9)
HL	19.1 ± 1.1 (17.3–20.9)	18.4 ± 1.3 (15.7–21)	18 ± 1.4 (15.9–18.9)	17.7	16.1 ± 0.6 (15.2–17.1)	14.7 ± 1.8 (12.7–16)
HW	12.3 ± 1.1 (10.5–14.1)	11.6 ± 1.1 (9.6–13.7)	11.6 ± 1.3 (9.9–13)	11.6	10.9 ± 0.6 (10.1–11.8)	10.2 ± 1 (9.1–11.1)
HH	7.7 ± 0.8 (6.4–9.1)	7.3 ± 0.8 (5.8–9)	7 ± 0.6 (6.2–7.4)	6.4	6.2 ± 0.8 (4.8–7.6)	6 ± 0.7 (5.4–6.8)
SL	7.8 ± 0.9 (6.6–9.3)	7.6 ± 0.6 (6.4–9)	7.2 ± 0.4 (6.7–7.7)	8.2	6.9 ± 0.4 (6.3–7.7)	6.5 ± 1.2 (5.2–7.3)
SW	10.1 ± 0.7 (8.8–11)	9.8 ± 0.8 (8.3–11.4)	9.6 ± 0.7 (8.7–10.3)	9.9	9 ± 0.3 (8.7–9.5)	8.5 ± 1.1 (7.3–9.3)
ED	5.1 ± 0.4 (4.5–5.8)	5 ± 0.3 (4.5–5.5)	4.9 ± 0.4 (4.3–5.4)	4.9	4 ± 0.2 (3.6–4.4)	3.8 ± 0.6 (3.2–4.4)
EVD	2.8 ± 0.3 (2.1–3.3)	2.5 ± 0.4 (1.9–3.2)	2.7 ± 0.4 (2.1–3.2)	2.1	1.9 ± 0.3 (1.3–2.3)	1.8 ± 0.5 (1.3–2.2)
LUn	12.3 ± 1.1 (9.8–13.8)	11.7 ± 1 (10.1–14.3)	10.2 ± 0.7 (9.3–10.8)	10	8.6 ± 0.4 (8–9.3)	7.6 ± 0.6 (6.9–8.2)
LHu	9.8 ± 0.8 (8.8–12.2)	9.5 ± 0.7 (8.4–11.1)	8.8 ± 0.7 (7.9–9.3)	8.7	7.5 ± 0.4 (6.7–8.2)	6.6 ± 0.5 (6.1–7)
LTb	15 ± 1.1 (12.4–16.8)	14.5 ± 1.1 (12.9–17.4)	13.1 ± 1 (12–14)	12.3	10.4 ± 0.6 (9–11.2)	10.1 ± 1.5 (8.4–11.3)
LFe	16 ± 1.1 (13–18)	15.3 ± 1.3 (12.6–17.8)	14.6 ± 1.3 (13–15.7)	13.1	12.1 ± 0.7 (10.5–13.1)	10.6 ± 1.2 (9.6–11.9)
Trow	13.9 ± 0.8 (12–15)	14 ± 1.1 (11–16)	15.3 ± 1 (14–16)	16	14 ± 1.3 (12–16)	13.3 ± 1.2 (12–14)
LT4	9.7 ± 0.7 (9–11)	9.7 ± 0.6 (9–11)	9.3 ± 0.5 (9–10)	8	8.1 ± 0.6 (7–9)	8.7 ± 0.6 (8–9)
ST4	3.4 ± 0.5 (3–4)	3 ± 0 (3)	3 ± 0 (3)	4	2.9 ± 0.3 (2–3)	3 ± 0 (3)
ULS	13.8 ± 1 (12–16)	13.7 ± 0.9 (12–15)	13.8 ± 1 (13–15)	13	13.3 ± 0.7 (12–14)	13.3 ± 1.2 (12–14)
LLS	10.4 ± 0.8 (9–12)	10.1 ± 0.8 (9–12)	10 ± 1.2 (9–11)	9	9.9 ± 0.6 (9–11)	10.3 ± 0.6 (10–11)

As linear body measurements are generally correlated with body size, all 12 morphometric variables (TrL, HL, HH, HW, SL, SW, ED, EVD, Lhu, Lun, Lfe and LTb) were regressed against SVL using ordinary least-squared regression in order to use the corresponding residues as a shape proxy. A principal component analysis (PCA) was then performed on the correlation matrix of the residuals to visualize the shape variation between the three species in a reduced dimensional space. In order to assess the contribution of each variable at segregating the species in the morphospace, a one-way ANOVA on each principal component was performed. Regarding body size, differences between groups were tested using a one-way ANOVA on the log-transformed values of SVL for each species pair. In addition, morphological differences between all species pairs were tested using a one-way ANOVA for each morphometric and meristic variable for taxonomic purposes (see taxonomic account). All data analysis and tests of significance were performed using the statistical software XLSTAT-Pro version 2008.6.8 (Addinsoft 1995–2008 software).

As a result of the low number of available vouchers, sexual dimorphism could only be tested for the larger of the two new species described herein, which is the only one with a proper sampling of both males and females (26 males and 17 females). Sexual dimorphism was tested using a one-way ANOVA for each variable. Summary statistics (mean, maximum, minimum and standard deviation) for each character of the three species formerly classified as *Asaccus caudivolvulus* were calculated for males and females independently (Table 1) and together (Table S2).

### Species concept, Zoobank registration and collection of specimens

In this manuscript we have adopted the General Lineage Species Concept (De Queiroz, 1998). This unified species concept considers species as separately evolving metapopulation lineages and treats this property as the single requisite for delimiting species. Other properties, such as phenetic distinguishability, reciprocal monophyly, and pre- and postzygotic reproductive isolation, are not part of the species concept but serve as important lines of evidence relevant to assess the separation of lineages and therefore to species delimitation (De Queiroz, 2007).

The electronic version of this article in Portable Document Format (PDF) will represent a published work according to the International Commission on Zoological Nomenclature (ICZN), and hence the new names contained in the electronic version are effectively published under that Code from the electronic edition alone. This published work and the nomenclatural acts it contains have been registered in ZooBank, the online registration system for the ICZN. The ZooBank LSIDs (Life Science Identifiers) can be resolved and the associated information viewed through any standard web browser by appending the LSID to the prefix http://zoobank.org/. The LSIDs for this publication is: urn:lsid:zoobank.org:pub:33DF71FE-3E0C-4907-8582-F7B369A0A713. The online version of this work is archived and available from the following digital repositories: PeerJ, PubMed Central and CLOCKSS.

Specimens were collected and manipulated with the authorization and under strict control and permission of the governments of Oman (Ministry of Environment and Climate Affairs, MECA) and the United Arab Emirates (Environment and Protected Areas Authority, Government of Sharjah). Specimens were captured and processed following the guidelines and protocols stated in the collecting permits and agreements obtained from the competent authorities of Oman and the United Arab Emirates (see references below). Members of the government supervised collecting activities. All efforts were made to minimize animal suffering. All the necessary collecting and export permits for this study in Oman were issued by the Nature Conservation Department of the Ministry of Environment and Climate Affairs, Oman (Refs: 08/2005; 16/2008; 38/2010; 12/2011; 13/2013; 21/2013) and the research in the United Arab Emirates was done under the supervision and permission of the Environment and Protected Areas Authority, Government of Sharjah.

## Results

### Molecular analyses

Dataset 1 consisted of a concatenated alignment of 2,310 base pairs (bp) for 11 individuals with 568 variable positions (*V*) and 397 parsimony informative sites (*Pi*) including the mitochondrial genes *12S* (402 bp; *V* = 185; *Pi* = 134) and *cytb* (399 bp; *V* = 217; *Pi* = 164) and the nuclear gene fragments *c-mos* (414 bp; *V* = 37; *Pi* = 25), *ACM4* (429 bp; *V* = 38; *Pi* = 21), and *MC1R* (666 bp; *V* = 91; *Pi* = 53). Dataset 2 consisted of a concatenated alignment of 2302 bp (*V* = 233; *Pi* = 222) including the mitochondrial genes *12S* (394 bp; *V* = 73; *Pi* = 69) and *cytb* (399 bp; *V* = 129; *Pi* = 125) and the nuclear gene fragments *c-mos* (414 bp; *V* = 12; *Pi* = 10), *ACM4* (429 bp; *V* = 7; *Pi* = 7), and *MC1R* (666 bp; *V* = 12; *Pi* = 11).

The results of the phylogenetic analyses of dataset 1 using BI and ML analyses produced similar trees with most of the nodes being well supported (Fig. 1B and Fig. S1). *Asaccus montanus* branches as a sister taxon to all the other *Asaccus* species included in the analysis. All three species from the Zagros Mountains (*A. elisae, A. griseonotus* and *A. nasrullahi*) form a very low supported clade and, within it, *A. griseonotus* and *A. elisae* are sister taxa. The Zagros endemics are sister taxa to a well-supported monophyletic group that includes the remaining species from the Hajar Mountains (*A. platyrhynchus*, *A. gallagheri*, *A. caudivolvulus* and the two new species described herein). Within this group, the three species formerly classified as *A. caudivolvulus* are recovered as an unsupported monophyletic group in the BI analysis (Fig. 1B) and are not monophyletic in the ML tree, in which the smaller of the two species described herein forms an unsupported group with the clade formed by *A. platyrhynchus* and *A. gallagheri* (Fig. S1).

**Table 2:** Uncorrected interspecific genetic distances between all *Asaccus* species included in the molecular phylogenetic analyses. (A) *p*-distances for *12S* mitochondrial gene (lower-left) and standard error estimates (upper-right). (B) *p*-distances for *cytb* mitochondrial gene (lower-left) and standard error estimates (upper-right).

		1	2	3	4	5	6	7	8	9
**(A)**										
1	*A. gardneri***sp. nov.**	–	0.012	0.018	0.019	0.018	0.019	0.021	0.018	0.019
2	*A. caudivolvulus*	0.068	–	0.018	0.02	0.019	0.019	0.021	0.019	0.019
3	*A. margaritae***sp. nov.**	0.147	0.162	–	0.021	0.017	0.02	0.021	0.019	0.017
4	*A. elisae*	0.192	0.218	0.209	–	0.02	0.016	0.021	0.019	0.02
5	*A. gallagheri*	0.168	0.18	0.157	0.206	–	0.02	0.02	0.019	0.018
6	*A. griseonotus*	0.196	0.203	0.213	0.116	0.211	–	0.02	0.02	0.021
7	*A. montanus*	0.221	0.235	0.216	0.223	0.204	0.218	–	0.02	0.02
8	*A. nasrullahi*	0.151	0.163	0.177	0.19	0.168	0.195	0.205	–	0.02
9	*A. platyrhynchus*	0.18	0.187	0.137	0.208	0.147	0.232	0.198	0.187	–

		1	2	3	4	5	6	7	8
**(B)**									
1	*A. gardneri***sp. nov.**	–	0.016	0.02	0.023	0.021	0.023	0.022	0.021
2	*A. caudivolvulus*	0.128	–	0.021	0.021	0.02	0.023	0.022	0.022
3	*A. margaritae***sp. nov.**	0.251	0.263	–	0.021	0.021	0.022	0.022	0.022
4	*A. gallagheri*	0.289	0.271	0.256	–	0.022	0.023	0.022	0.022
5	*A. griseonotus*	0.225	0.23	0.238	0.256	–	0.022	0.022	0.021
6	*A. montanus*	0.308	0.301	0.275	0.316	0.274	–	0.023	0.023
7	*A. nasrullahi*	0.265	0.26	0.258	0.297	0.245	0.278	–	0.024
8	*A. platyrhynchus*	0.253	0.256	0.263	0.273	0.256	0.308	0.305	–

**Figure 2:**
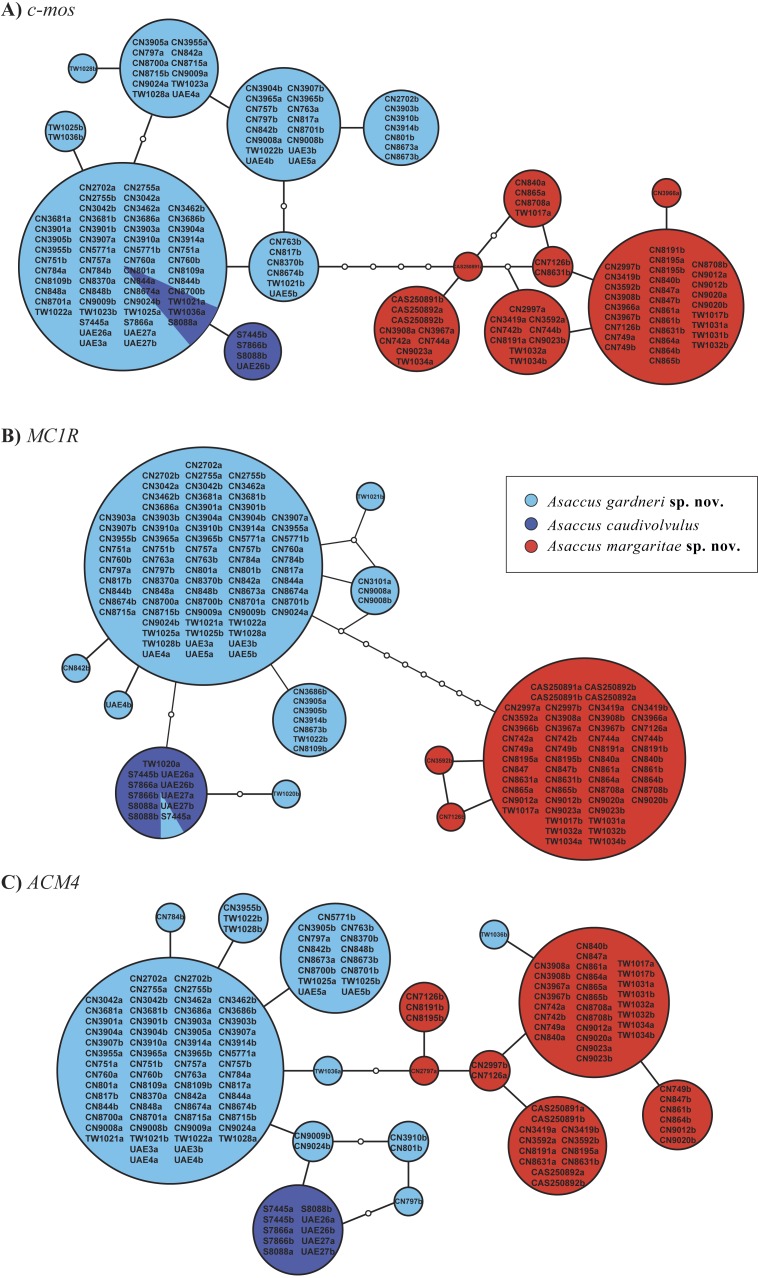
Statistical parsimony nuclear allele networks. (A) *c-mos*, (B) *MC1R*, (C) *ACM4*. Circle sizes are proportional to the number of individuals and white circles represent mutational steps. Phase probabilities were set as ≥0.7. Detailed information on the samples included in the network analysis is given in Table S1.

**Figure 3:**
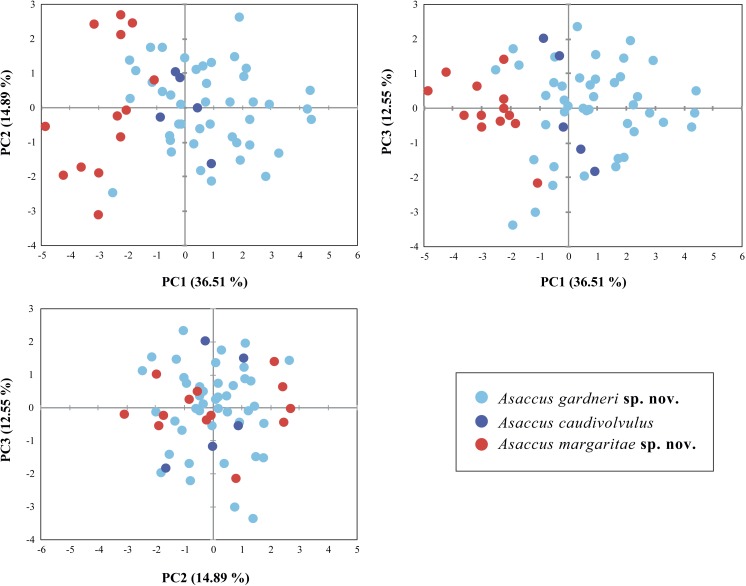
Principal Component Analysis (PCA) of the shape related morphological data. The contribution of the first three principal components is given in brackets. See material and methods and Table S3 for more details.

Genetic distances between all *Asaccus* species included in our analyses are very high, varying between 6.8%–23.2% for the *12S* and 12.8%–30.8% for the *cytb* (Table 2). The smallest genetic distance between two species corresponds to the comparison between *A. caudivolvulus* and the larger of the two species described herein and the wide genetic distance between two species corresponds to the comparison between *A. platyrhynchus* and *A. griseonotus*. The level of intraspecific genetic variability for the smaller of the two species described herein is 0.12 ± 0.08% for the *12S* and 0.38 ± 0.17% for the *cytb*; intraspecific genetic variability for the larger of the two species described herein is 0.33 ± 0.16% for the *12S* and 0.87 ± 0.26% for the *cytb*, and intraspecific genetic variability for *A. caudivolvulus* is 0% for both mitochondrial genes.

Inferred ages from the phylogeny are shown in Fig. 1B and indicate that diversification in *Asaccus* started at least 24.6 Ma (95% HPD = 15.2–38.4). The clade formed by *A. gallagheri*, *A. platyrhynchus*, *A. caudivolvulus* and the two new species described herein started diversifying 14.2 Ma (95% HPD = 8.8–21.9) and, within it, divergence between the three species formerly classified as *A. caudivolvulus* started 12.7 Ma (95% HPD = 7.6–19.8) and divergence between the larger of the two species described herein and *A. caudivolvulus* occurred 4.0 Ma (95% HPD = 2.1–6.7).

The results of the phylogenetic analyses of dataset 2 assembled to study in detail the phylogeographic relationships of *Asaccus caudivolvulus* and the two new species described herein are shown in Fig. 1C. Despite very good sampling across the northern Hajar Mountains, the variability within each one of the three lineages is very low. Like in Fig. 1B, all three lineages are phylogenetically very well differentiated.

The results of the haplotype network analyses are presented in Fig. 2 and clearly show that the smaller of the two species described herein does not share a single allele in all three nuclear genes analysed with any of the other two species of the *A. caudivolvulus* radiation. Regarding *A. caudivolvulus* and the larger of the two species described herein, they only share one allele in the *MC1R* gene and two alleles in the *c-mos* gene. As a result of the reduced geographical (one locality only) and numerical (five specimens) sampling, *Asaccus caudivolvulus* only presents four haplotypes (two in the *c-mos*, one in the *MC1R* and one in the *ACM4* genes). In contrast, as a result of the relatively high number of specimens analysed, the small and large species described herein present 16 and 25 haplotypes, respectively (see Fig. 2). The different haplotypes of the two new species described herein do not present any geographic structure, being distributed evenly over the various sampling sites (see Fig. 1A, Fig. 2 and Table S1). As shown in the three independent phylogenetic trees inferred using the phased nuclear datasets (Fig. S2), none of the three species of the *A. caudivolvulus* radiation (*A. caudivolvulus* and the two species described herein) share any alleles with other *Asaccus* species from Fig. 1B.

### Multivariate analysis of morphological data

Shape differences between the two new species described herein and *A. caudivolvulus* are shown in Fig. 3 and descriptive statistics for all 18 characters are presented in Table 1 and Table S2. The results of the sexual dimorphism analysis showed that there are significant differences in size (SVL) (*F* = 5.188; d.f. = 1; *P* = 0.028) but not in the other morphometric characters. As a result, in the analyses both sexes were pooled together to test shape differences but size was treated as a dimorphic character. As a result of the low number of females, size differences between species were only tested among male specimens using a one-way ANOVA on the log-transformed values of SVL. The result of this test was highly significant in the comparison between the smaller and the larger of the two new species described herein (*F* = 49.434; d.f. = 1; *P* = <0.0001) and also in the comparison between the smaller of the two new species described herein and *A. caudivolvulus* (*F* = 10.304; d.f. = 1; *P* = 0.007). Shape differences between species were tested with one-way ANOVA on the PCA scores of the 12 components and the result was significant for the first component (*P* = <0.0001), which represents one third of the total variability. The highest loadings in this component are the four limb characters (Lhu, Lun, Lfe and LTb) (Table S3). These results indicate that the smaller species of the *A. caudivolvulus* radiation presents lower values for all characters related to the length of the extremities (corrected for SVL, see Material and Methods) compared with the other two species of *Asaccus* from which it segregates clearly in the first axis of the PCA (Fig. 3). The results of the PCA analysis of body shape summarized in Fig. 3 also show that the larger of the two species described and *Asaccus caudivolvulus* overlap in the morphospace. Differences in the head (HL) and the eye diameter (ED) between the smaller and the larger of the two new species described are also remarkable, despite having a lower contribution than the length of the extremities in the first axis of the PCA (see Tables S3, S4 and taxonomic section).

### Taxonomic section

**Figure 4:**
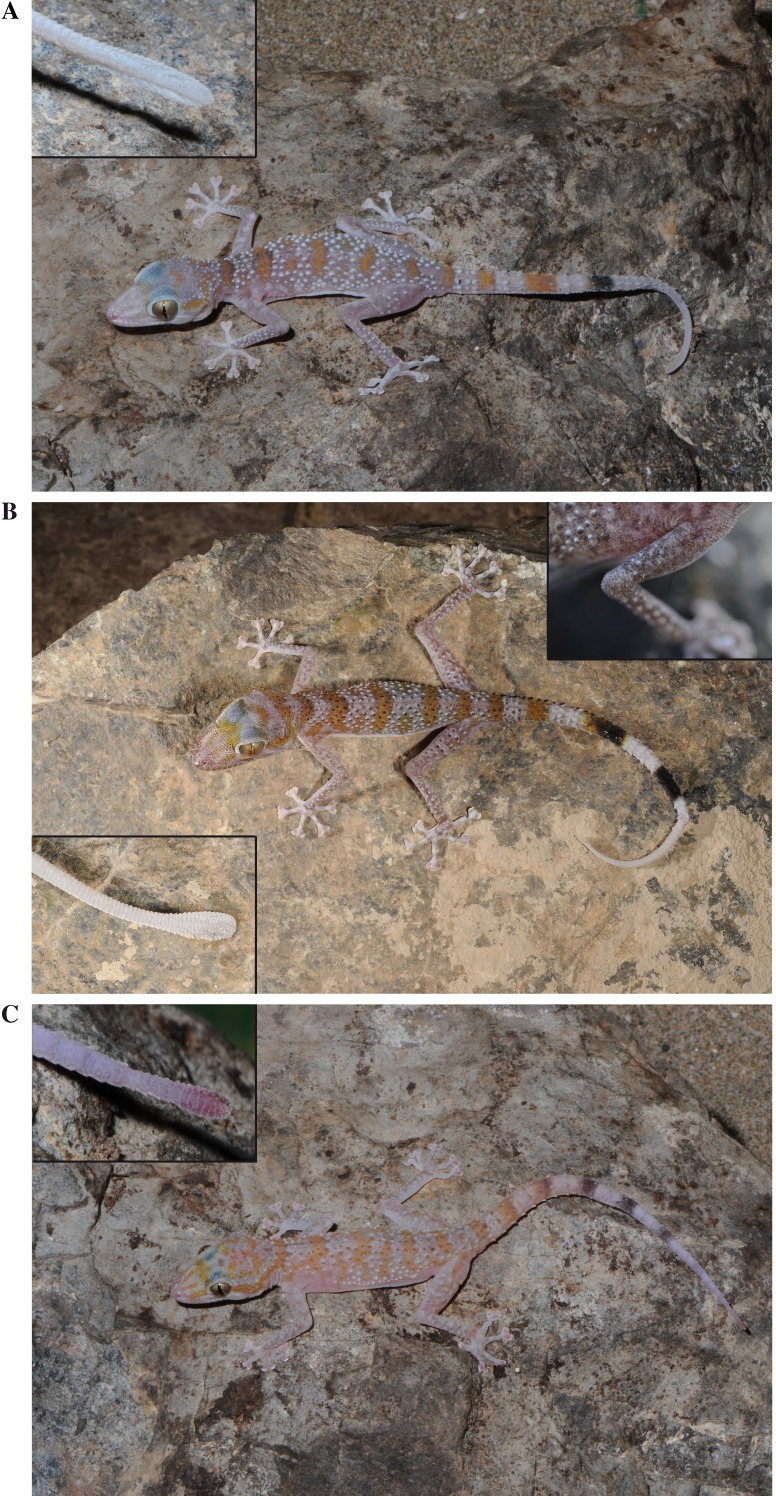
General appearance and colour in life of the three species studied in this work. (A) Holotype of *Asaccus gardneri* **sp. nov.** (voucher code BMNH2008.1000) including a detail of the tail tip; (B) *Asaccus caudivolvulus* (specimen not collected) including a detail of the tail tip and of the tubercles present on the upper arm; (C) Holotype of *Asaccus margaritae* **sp. nov.** (voucher code BMNH2008.989) including a detail of the tail tip.

**Figure 5:**
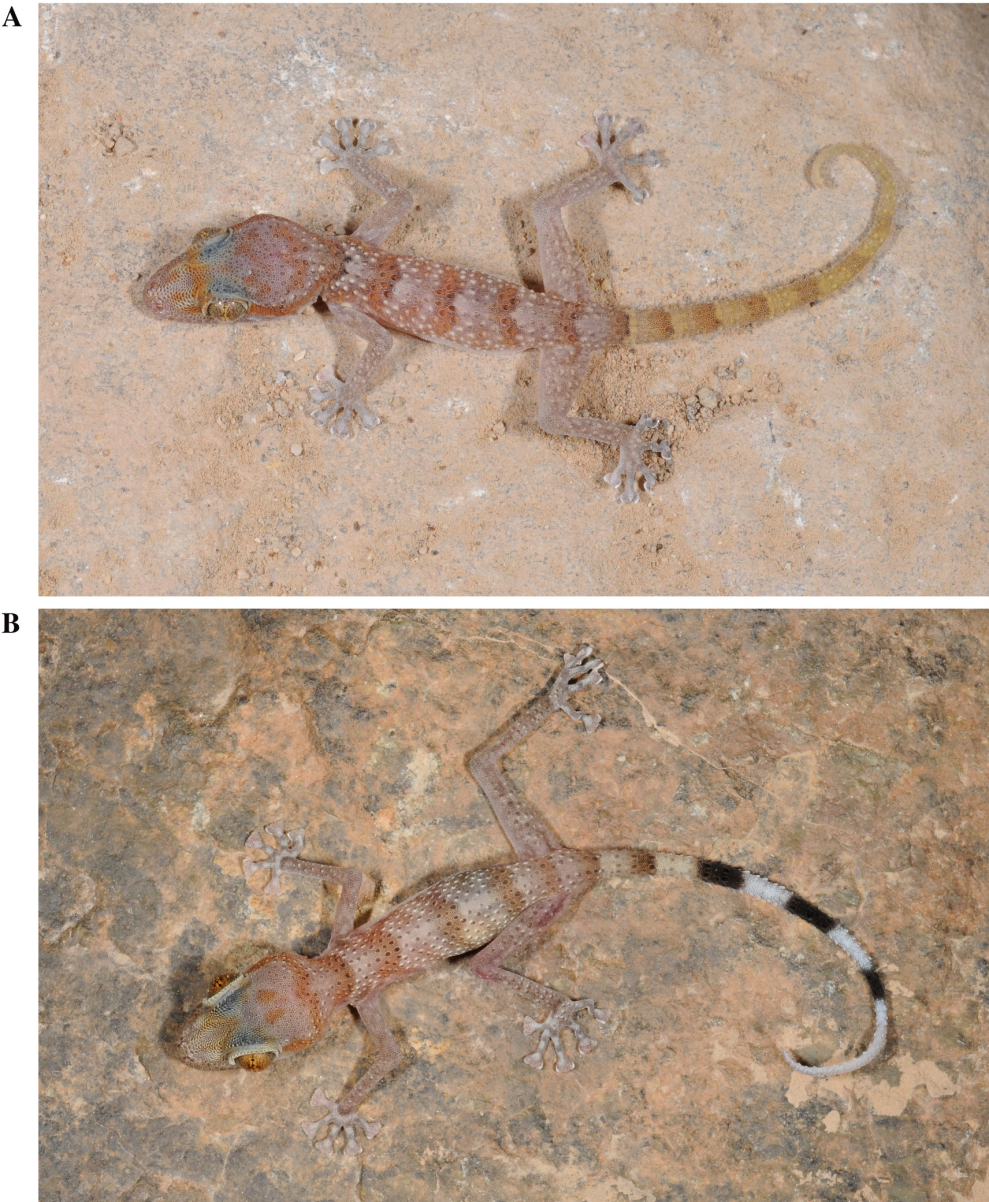
Pictures of juvenile specimens. (A) *Asaccus margaritae* **sp. nov.** (B) *Asaccus caudivolvulus*. Juveniles of *Asaccus gardneri* **sp. nov.** are very similar to juveniles of *Asaccus caudivolvulus* (S Carranza & J Els, pers. obs., 2013).

Given the genetic distinctiveness of the three lineages of *Asaccus* previously classified as *A. caudivolvulus* from the northern Hajar Mountains in the two mitochondrial and three nuclear gene fragments analysed (Figs. 1–2; Table 2) and the morphological differences (see diagnosis below, Figs. 3–5 and Tables S2–S4), we describe the two unnamed lineages as new species. The third genetic lineage is composed of specimens from locality 29 in Figs. 1A and 1C; very near the type locality of *Asaccus caudivolvulus* (locality 31 in Figs. 1A and 1C). Despite the fact that genetic material could not be obtained from the type locality of *A. caudivolvulus* as a result of heavy development and inaccessibility to the area, all the specimens from locality 29 that have been included in the genetic analyses share with the type specimens of *A. caudivolvulus* from locality 31 the presence of small tubercles on the upper arms, a character not present in the other two species of the *A. caudivolvulus* species complex described herein. As a result of the presence of this unambiguous character, we can confidently assign all the specimens from locality 29 to the species *A. caudivolvulus*. Data for the morphological description of the two new species was obtained from our own morphological dataset (Table S2) and also from morphological information available from the original descriptions of all 16 species of *Asaccus* (Arnold, 1972; Dixon & Anderson, 1973; Arnold & Gardner, 1994; Gardner, 1994; Rastegar-Pouyani, 1996; Rastegar-Pouyani, Nilson & Faizi, 2006; Werner, 2006; Afrasiab & Mohamad, 2009; Torki, 2010; Torki et al., 2011a; Torki et al., 2011b).

**Family Phyllodactylidae**

**Genus *Asaccus*** Dixon & Anderson, 1973

**Figure 6:**
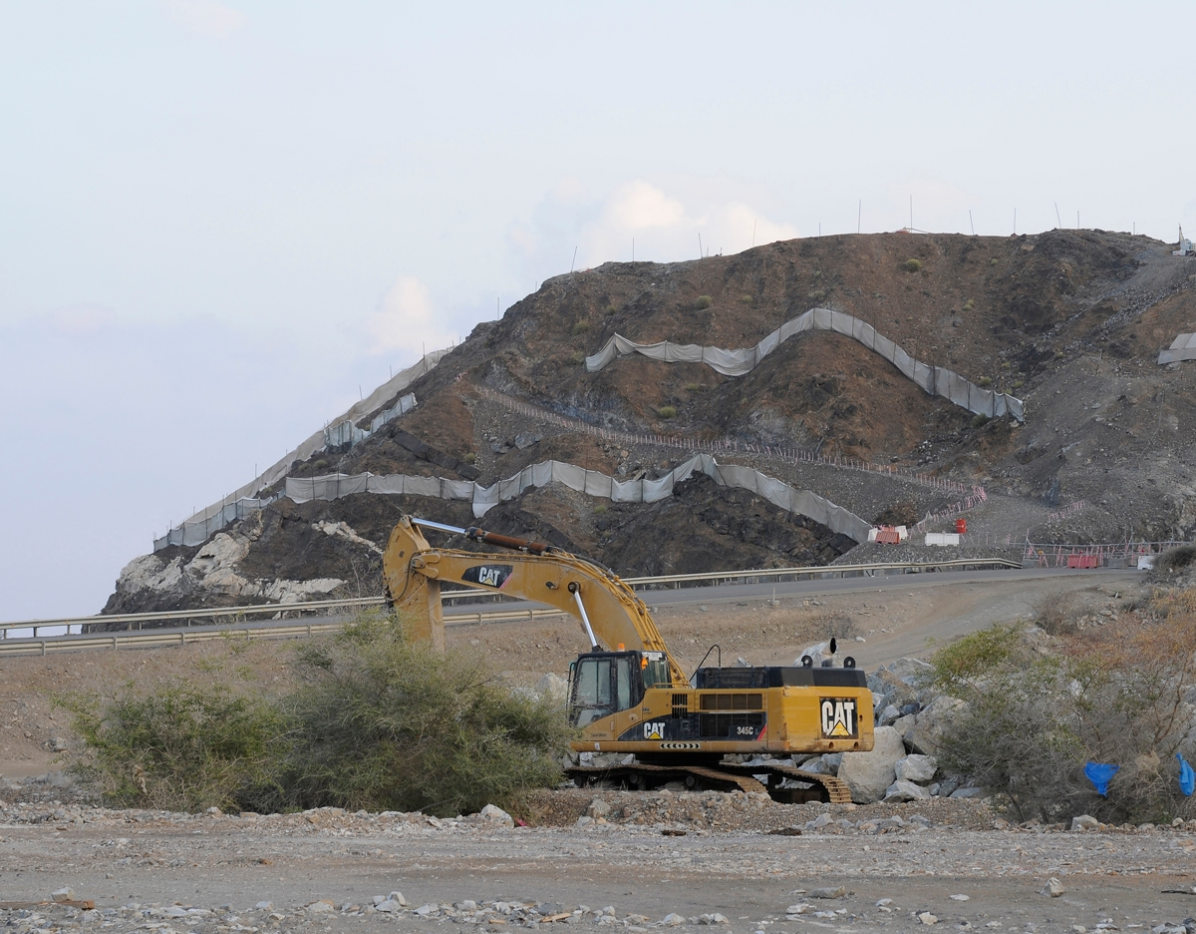
Locality of *Asaccus caudivolvulus* under heavy transformation. View of locality 29 (Fig. 1A) on the 1st of December 2015. The little mountain by the sea is the only locality where *Asaccus caudivolvulus*, the only endemic vertebrate of the UAE, has been recorded since 1994.

***Asaccus caudivolvulus*** Arnold & Gardner, 1994

(Figs. 1–6, Fig. S1; Tables 1–2, Tables S1–S4)

*Holotype*. BMNH1973.1850, adult male, Jebel Ra’s, 2.5 km South of Khawr Fakkan (UAE), elevation 186 m a.s.l., collected by E. N. Arnold and M. D. Gallagher on the 5th of May 1973.

*Paratype*. BMNH1973.1851, adult male, same data as holotype.

*Other material examined*. Two specimens used only for genetic analyses (no voucher available) and three specimens used for genetic and morphological analyses; all listed in Table S1.

*Diagnosis.* A species of *Asaccus* endemic to the United Arab Emirates characterized by the combination of the following characters: (1) medium size (up to 63.2 mm from snout to vent); (2) fine scales across supraorbital region; (3) relatively large limbs; (4) two pairs of postmentals, first in contact; (5) keeled trihedral moderate-sized dorsal tubercles present on back (14–16 longitudinal rows at mid-body); (6) small pointed tubercles on occiput, neck and sides of head; (7) keeled tubercles present on forearms and hind limbs; (8) small tubercles present on upper arms (Fig. 4B); (9) paired terminal scansors on digits extending well beyond claws; (10) cloacal tubercles small; (11) subcaudal series of expanded scales reaching vent area anteriorly, (12) tail tip laterally compressed and vertically expanded (Fig. 4B); (13) dorsum with a pattern of approximately 5 orange-brown transverse bars (one on neck, three on body and one on sacrum; Fig. 4B); (14) tail colour not sexually dimorphic; (15) adults with whitish-ivory tails (whiter distally) with 4-5 wide orange-dark transverse bands (last 2–3 crossbands black and extending ventrally) (Fig. 4B); (16) tail can be coiled and waved.

Proposal of common names:

English: Emirati Leaf-toed Gecko

Arabic: الوزغةالإماراتيةورقيةالأصابع

***Asaccus gardneri*** **sp. nov.**

urn:lsid:zoobank.org:act:0532F92E-5136-4E0C-A752-B4973590308D

(Figs. 1–4, Figs. S1–S2; Tables 1–2, Tables S1–S4)

*Asaccus caudivolvulus.* Arnold & Gardner, 1994: 426, 431 (part.); Van der Kooij, 2000: 107 (part.); Sindaco & Jeremcenko, 2008: 98 (part.); Papenfuss et al., 2010: 586 (part); Torki et al., 2011a: 1 (part); Gardner, 2013: 91 (part.);

*Holotype*. BMNH2008.1000, adult male, from Musandam Peninsula (Oman), 26.14934N 56.16193E WGS84, elevation 16 m a.s.l., collected by S. Carranza, M. Metallinou, Ali Alghafri, Sultan Khalifa and Hamed Al Furkani on the 21st of April 2013 between 23:15–23:45, tissue code CN3905.

*Paratypes.* BMNH2008.999 and ONHM4221, two adult females, from Musandam Peninsula (Oman), 26.21208N 56.23555E WGS84, elevation 17 m a.s.l., collected by S. Carranza, M. Metallinou, Ali Alghafri, Sultan Khalifa and Hamed Al Furkani on the 21st of April 2013 between 21:10–21:40, tissue codes CN5771 and CN2755, respectively; IBECN751, adult male, and IBECN844, adult female both from Musandam Peninsula (Oman), 26.22759N 56.21372E WGS84, elevation 5 m a.s.l., collected by S. Carranza, M. Metallinou, Ali Alghafri, Sultan Khalifa and Hamed Al Furkani on the 21st of April 2013 between 22:10–22:40, tissue codes CN751 and CN844, respectively; IBECN10423, IBECN10424 and IBECN10425, three adult females and IBECN10426 and IBECN10427, two adult males, all from Wadi Beh (Oman), 25,746417N 56,278278E WGS84, elevation 280 m a.s.l., collected by J. Els, S. Jayasinghe and T. Wilms on the 13th of February 2012 between 20:00–21:00, tissue codes, TW1020, TW1023, TW1036, TW1021 and TW1022, respectively; IBECN10428, adult female, from Ras al-Kaimah (UAE), 25,613917N 56,029556E WGS84, elevation 200 m a.s.l., collected by J. Els, S. Jayasinghe and T. Wilms on the 14th of February 2012 between 21:00–23:00, tissue code TW1028; BMNH1976.1414-15 and BMNH1976.1419, three adult females and BMNH1975.1416-1418, three adult males, all from Khasab (Oman), elevation 8 m a.s.l., collected by M. D. Gallagher on the 5th of November 1975.

*Other material examined*. Two specimens used only for morphological analyses (museum specimens not available for DNA analyses), 14 used only for genetic analyses (no voucher available, juvenile or damaged specimen) and 24 used for genetic and morphological analyses; all listed in Table S1.

*Etymology.* The species epithet “*gardneri”* is a genitive Latin noun to honor the British herpetologist Dr. Andrew S. Gardner for his life-long dedication and contribution to Arabian herpetology. He was the first to highlight, together with E. N. Arnold, the distinctiveness of *Asaccus gardneri* **sp. nov.** (*Asaccus caudivolvulus* Khasab population *sensu* Arnold & Gardner, 1994).

*Diagnosis.* A new species of *Asaccus* from Oman and the United Arab Emirates characterized by the combination of the following characters: (1) large size (up to 70.7 mm from snout to vent); (2) relatively large limbs; (3) two pairs of postmentals, first in contact; (4) fine scales across supraorbital region; (5) keeled trihedral moderate-sized dorsal tubercles present on back (11–16 longitudinal rows at mid-body); (6) small pointed tubercles on occiput, neck and sides of head; (7) keeled tubercles present on forearms and hind limbs but absent on upper arms; (8) paired terminal scansors on digits extending well beyond claws; (9) cloacal tubercles small; (10) subcaudal series of expanded scales reaching vent area anteriorly; (11) tail tip laterally compressed and vertically expanded (strongly expanded in some specimens) (Fig. 4A); (12) dorsum with a pattern of approximately 5 orange-brown transverse bars (one on neck, three on body and one on sacrum; Fig. 4A); (13) tail colour not sexually dimorphic; (14) adults with whitish-ivory tails (whiter distally) with 3–5 wide orange-dark transverse crossbands (last 1–2 crossbands black and extending ventrally) (Fig. 4A); (15) tail can be coiled and waved.

*Differential diagnosis*. *Asaccus gardneri* **sp. nov.** differs from its closely related taxon *A. caudivolvulus* mainly in its larger size (SVL max. 70.7 mm, compared with max. 63.2 mm) and in the absence of tubercles on the upper arm (tubercles on upper arm present on *A. caudivolvulus*). It further differs in having fewer distal black crossbands on tail that extend ventrally (1–2 *versus* 2–3), in having fewer longitudinal rows of tubercles at mid-body on back (11–16 *versus* 14–16; ANOVA comparison of Trow significant, *P* < 0.01; Table S4), by a genetic distance of 12.8% and 6.8% in the mitochondrial *cytb* and *12S* genes, respectively (Table 2), and by the presence of different alleles in the *acm4* nuclear gene analysed here (Fig. 2). It differs from the other new species of *Asaccus* from Oman and UAE described herein mainly in its larger size (SVL max. 70.7 mm, compared with max. 58.6 mm), in having longer fore limbs and hind limbs with respect to SVL (ANOVA comparisons of LUn, LHu, LTb and LFe all significant, *P* < 0.0001; Table S4), in the different tail colour in juveniles (white tail *versus* conspicuously orange-coppery tail; Fig. 5A), in the size of the tubercles on the occiput, neck and sides of head (small size tubercles *versus* large pointed tubercles). It further differs in having a longer head (ANOVA comparison of HL significant, *P* < 0.01; Table S4), in having larger eyes (ANOVA comparison of ED significant, *P* < 0.0001; Table S4), in having more lamella under the fourth toe (9–11 *versus* 7–9; ANOVA comparison of LT4 significant; *P* < 0.0001; Table S4), in having fewer distal black crossbands on the tail that extend ventrally (1–2 *versus* 2–3), by a genetic distance of 25.1% and 14.6% in the mitochondrial *cytb* and *12S* genes, respectively and in the presence of different alleles in the *acm4*, *cmos* and *mc1r* nuclear genes analysed here. It differs from *Asaccus andersoni* in its larger size (SVL max. 70.7 mm, compared with max. 66.2 mm), in the number and disposition of the postmentals (2 pairs of postmentals, the first pair in contact *versus* 2–3 pairs of postmentals, first pair separated by mental), in the presence of crossbands on the tail that extend ventrally (crossbands on tail do not extend ventrally in *A. andersoni*), in the size and shape of the tubercles on dorsal body (keeled trihedral moderate-sized dorsal tubercles *versus* smooth tubercles), in the absence of sexual dichromatism (presence in *A. andersoni*). It differs from *Asaccus barani* in its larger size (SVL max. 70.7 mm, compared with max. 56 mm), in the paired terminal scansors on the digits (extend well beyond claws *versus* do not extend beyond claws), in the tubercles on the upper arm (absent *versus* present). It differs from *Asaccus elisae* in its larger size (SVL max. 70.7 mm, compared with max. 57.9 mm), in the scales across the supraorbital region (fine *versus* coarse), in the absence of tubercles on the upper arm (tubercles on upper arm present on *A. elisae*), in the paired terminal scansors on digits (extend well beyond claws *versus* do not extend beyond claws), in the ability to coil the tail laterally like a loose watch-spring (inability to curl the tail in *A. elisae*) and by a genetic distance of 19.2% in the mitochondrial *12S* gene. It differs from *Asaccus gallagheri* in its larger size (SVL max. 70.7 mm, compared with max. 40 mm), in the presence of dorsal tubercles on the back, occiput, and elsewhere (absence of tubercles in *A. gallagheri*), in the tail tip laterally compressed and vertically expanded (tail tip not laterally compressed or vertically expanded in *A. gallagheri*), in the tail colour not being sexually dimorphic (tail colour sexually dimorphic in *A. gallagheri*; white with black crossbars in females and yellow in males), in the ability to coil the tail laterally like a loose watch-spring (inability to curl the tail in *A. gallagheri*) and by a genetic distance of 28.9% and 16.8% in the mitochondrial *cytb* and *12S* genes, respectively. It differs from *Asaccus granularis* in the presence of tubercles on the occiput, neck and sides of head (absence in *A. granularis*), in the presence of 1–2 crossbands on the tail extending ventrally (crossbands on tail do not extend ventrally in *A. granularis*), in the ability to coil the tail laterally like a loose watch-spring (inability to curl the tail in *A. granularis*). It differs from *Asaccus griseonotus* in the scales across the supraorbital region (fine *versus* coarse), in the dorsal tubercles (keeled trihedral moderate-sized dorsal tubercles present on back and small pointed tubercles on occiput, neck and sides of head *versus* small dorsal tubercles present on back and tubercles absent on occiput), in the presence of 1–2 crossbands on the tail extending ventrally (crossbands on tail do not extend ventrally in *A. griseonotus*), in the ability to coil the tail laterally like a loose watch-spring (inability to curl the tail in *A. griseonotus*) and by a genetic distance of 22.5% and 19.6% in the mitochondrial *cytb* and *12S* genes, respectively. It differs from *Asaccus iranicus* in its larger size (SVL max. 70.7 mm, compared with max. 41.8 mm), in the fore limb fingers not being parallel to the arm (parallel to the arm in *A. iranicus*; Figs. 3–4 of Torki et al., 2011a), in the paired terminal scansors on digits (extend well beyond claws *versus* do not extend beyond claws), in the absence of tubercles on the upper arm (tubercles on upper arm present on *A. iranicus*). It differs from *Asaccus kermanshahensis* in its larger size (SVL max. 70.7 mm, compared with max. 55.7 mm), in the number of postmentals (2 pairs *versus* 4 pairs), in the dorsal tubercles (keeled trihedral moderate-sized dorsal tubercles present on back and small pointed tubercles on occiput, neck and sides of head *versus* smooth oval or round dorsal tubercles present on occiput, neck and sides of head), in the presence of 1–2 crossbands on tail extending ventrally (tail without dark rings in *A. kermanshahensis*). It differs from *Asaccus kurdistanensis* in its larger size (SVL max. 70.7 mm, compared with max. 63.5 mm), in the number of postmentals (2 pairs *versus* 3 pairs), in the dorsum colouration (dorsum pale pink with a pattern of approximately 5 orange-brown transverse bars -one on neck, three on body and one on sacrum- *versus* dorsum whitish-grey with dark large spots scattered throughout), in the number of transverse bands on the tail (3–5 *versus* more than 5; up to 9–10 according to Rastegar-Pouyani, Nilson & Faizi, 2006). It differs from *Asaccus montanus* in its larger size (SVL max. 70.7 mm, compared with max. 40 mm), in the dorsal tubercles (keeled trihedral moderate-sized dorsal tubercles present on back and small pointed tubercles on occiput, neck and sides of head *versus* very large keeled dorsal tubercles on back -some in contact-, occiput and sides of head), in the absence of tubercles on the upper arm (very large keeled tubercles on upper arm present in *A. montanus*), in the paired terminal scansors on the digits (extend well beyond claws *versus* do not extend beyond claws) and by a genetic distance of 30.8% and 22.1% in the mitochondrial *cytb* and *12S* genes, respectively. It differs from *Asaccus nasrullahi* in the scales across the supraorbital region (fine *versus* coarse), in the dorsal tubercles (keeled trihedral moderate-sized dorsal tubercles present on back in 11-16 longitudinal rows at mid-body and small pointed tubercles on occiput, neck and sides of head *versus* small, circular tubercles on back in fewer than 9 longitudinal rows at mid-body and absence of tubercles on occiput and sides of head) and by a genetic distance of 26.5% and 15.1% in the mitochondrial *cytb* and *12S* genes, respectively. It differs from *Asaccus platyrhynchus* in its larger size (SVL max. 70.7 mm, compared with max. 63 mm), in the dorsal tubercles (keeled trihedral moderate-sized dorsal tubercles present on back *versus* small tubercles on back), in the tail tip laterally compressed and vertically expanded and tail colour not being sexually dimorphic (tail tip not laterally compressed or vertically expanded and tail colour sexually dimorphic -white with black crossbars in females and yellow in males- in *A. platyrhynchus*) and by a genetic distance of 25.3% and 18% in the mitochondrial *cytb* and *12S* genes, respectively. It differs from *Asaccus saffinae* in its larger size (SVL max. 70.7 mm, compared with max. 57 mm), in the dorsal tubercles (keeled trihedral moderate-sized dorsal tubercles present on back and small pointed tubercles on occiput, neck and sides of head *versus* smooth, unkeeled dorsal tubercles present on back, a few very small tubercles on occiput and no tubercles on head), first pair of postmentals in contact (not in contact in *A. saffinae*). It differs from *Asaccus tangestanensis* in its larger size (SVL max. 70.7 mm, compared with max. 57 mm), in the absence of tubercles on the upper arm (tubercles on upper arm present in *A. tangestanensis*), in the paired terminal scansors on digits (extend well beyond claws *versus* do not extend beyond claws). It differs from *Asaccus zagrosicus* in its larger size (SVL max. 70.7 mm, compared with max. 55 mm), in the absence of tubercles on the upper arm (tubercles on upper arm present on *A. zagrosicus*), in the paired terminal scansors on the digits (extend well beyond claws *versus* do not extend beyond claws), in the secondary postmentals (in contact with lower labials *versus* separated from lower labials by 1–3 rows of scales).

*Description of the holotype.* BMNH2008.1000. (Fig. 4A). Complete specimen with the tip of the tongue missing (used for DNA extraction). Data on 13 morphometric and five meristic variables (see Material and Methods) are provided in Table S2. Adult male, SVL 63.4 mm, depressed head and body, well-marked neck, relatively slender limbs and tail, snout strongly depressed with a concave upper profile in lateral view. Head length 29% of SVL and head width 72% of head length. Tail not regenerated, 1.21 times SVL. Rostral scale rectangular, with upper edge with shallow W shape, more than twice as wide as high and without a median cleft. Nostril bordered by rostral, first labial, internasal and two postnasal scales, all entering into its border, the two internasal scales in contact behind the rostral. Three distinct depressions exist, one right behind the nostrils, another one right in front of the lower part of the eyes and the last ones in the area comprised between the eyes down to the nostrils. Snout area with flat scales and no tubercles, eyes large, diameter about 26% of head length, vertical pupil, palpebral fold edged anteriorly with enlarged imbricated scales that decrease in size towards the back and become ciliated, one row of supraorbital rounded tubercles. Ear opening vertical (elliptical), smooth edged, approximately twice longer than wide. Mental scale large, wedge-shaped, extending backwards to the level of the sutures between second and third lower labial scales; two pairs of postmental scales, the first pair in contact with first and second lower labials and larger than the second pair, which is only in contact with the second lower labials; first pair of postmentals in broad contact and extending backwards to the level of the sutures between third and fourth lower labial scales; gular scales flat and small, without tubercles. Dorsum covered with uniform, flattened granules with rounded outline, interspersed with moderate-sized keeled trihedral tubercles; small pointed tubercles on occiput, neck and sides of head; keeled tubercles present on forearms and hind limbs but absent on upper arms; ventral scales flat, slightly imbricate and about twice the diameter of dorsal scales at mid-belly, larger in the pelvic region. Underside of digits with enlarged scales (10 under the 4th digit) that become replaced by irregular transverse rows of smaller scales (four under the 4th digit); paired terminal scansors on digits longer than wide, projecting well beyond claws. Tail distinctly dorsoventrally flattened at base, but round distally, tail tip laterally compressed and vertically expanded; tail divided in segments covered above by small slightly imbricate scales and six large keeled trihedral tubercles on the basal segments, reduced to four smaller ones distally; underside of tail with a single series of expanded approximately rectangular scales, two per segment, reaching the vent area anteriorly.

Colouration in alcohol whitish-yellow underneath and brownish with interspersed small white speckles, with five faint irregular transversal dark crossbands across the back (one on neck, three on body and one on sacrum). Tail with five dark crossbands increasing in intensity distally, the last one not clearly visible above; ventral surface of tail pale with the two distal most crossbands extending into it very strongly. Colour in life much richer than in the fixed specimen (Fig. 4A), pale pink with all the above-described pattern of marks more evident, with the five dorsal and first three tail crossbands orange, and the two distalmost tail crossbands black. Faint orange stripes present on the head area. Iris in life colourful, golden with dark venations.

*Variation.* Data on 13 morphometric and five meristic variables (see Material and Methods) for all 10 paratypes (see above) are provided in Table S2. Specimens IBECN10423, IBECN10427, IBECN10424, IBECN10428 and IBECN10425 all with regenerated tails and specimen IBECN10426 with half of the tail missing. All five paratypes with an incision on the ventral side of the thigh from where muscular tissue was removed for DNA extraction. Specimens BMNH2008.999, IBECN844 and IBECN751 with regenerated tails, ONHM4221 with broken tail; in all four specimens the tip of the tongue was cut and used for DNA extraction. All the specimens are very similar to each other, main colouration very similar to the holotype, with paratypes IBECN10423-28 darker and with more conspicuous crossbands.

*Distribution and ecology.* As a result of the intensive sampling across the Hajar Mountain range carried out between 2004 and 2016, *Asaccus gardneri* **sp. nov.** has been found well distributed from the Musandam Peninsula (Khasab), Oman, to latitude 25.30012 N in the UAE (Loc. 32 in Fig. 1A; Table S1). It is an endemic species to the northernmost part of the Hajar Mountain range.

*Asaccus gardneri* **sp. nov.** has been found on rocky sides of wadis, stony substrates, lower down on large boulders, and hiding in the caves. Strictly nocturnal, all specimens were captured during the night and avoided the beam of the flashlight, running into crevices and holes and fleeing across boulders with incredible agility and ease. The lizards can be very common in some areas.

*Conservation status.* Not evaluated.

Proposal of common names:

English: Gardners’ Leaf-toed Gecko

Arabic: وزغةغاردنرورقيةالأصابع

***Asaccus margaritae*** **sp. nov.**

urn:lsid:zoobank.org:act:F6966A21-28A1-4A36-92E9-B56833144C29

(Figs. 1–5; Figs. S1–S2; Tables 1–2; ; Tables S1–S4)

*Holotype*. BMNH2008.989, adult male, from Wadi Al Helo (UAE), 25.00768N 56.21518E WGS84, elevation 373 m a.s.l., collected by J. Els, S. Jayasinghe, S. Carranza and M. Metallinou on the 24th of April 2013 between 21:00–23:00, tissue code CN3966.

*Paratypes.* BMNH2008.988 and ONHM4222, two females and IBECN9012, IBECN9023 and IBECN8708, three adult males, all five specimens with same data as Holotype, tissue codes CN3967, CN3908, CN9012, CN9023 and CN8708, respectively; IBECN10419, IBECN10420, IBECN10421, three adult males and IBECN10422, an adult female, all four specimens from Wadi Al Helo (UAE), 25,006861N 56,215556E WGS84, elevation 374 m a.s.l., collected by J. Els, S. Jayasinghe and T. Wilms on the 27th of July 2010 between 21:30–23.00, tissue codes TW1017, TW1031, TW1032 and TW1033, respectively.

*Other material examined*. Sixteen specimens used only for genetic analyses (no voucher available, juvenile or damaged specimens) and three specimens used for genetic and morphological analyses; all listed in Table S1.

*Etymology.* The species epithet “*margaritae”* is a genitive Latin noun to honor the Greek scientist Dr. Margarita Metallinou whose career was tragically cut short by a wildlife accident while doing fieldwork in Africa on the 2nd of July 2015. This new species is a special tribute to her memory from all the authors of this manuscript, with whom she shared many unforgettable moments both in Arabia and in Barcelona. It is also a recognition of her enthusiasm, passion and dedication to herpetology and especially for her work on Arabian geckos, including the genus *Asaccus* (Carranza & Bauer, 2016).

*Diagnosis.* A new species of *Asaccus* from Oman and UAE characterized by the combination of the following characters: (1) medium size (up to 58.7 mm from snout to vent); (2) relatively short limbs; (3) two pairs of postmentals, first in contact; (4) scales across supraorbital region fine with sparse scattered pointed tubercles; (5) keeled trihedral moderate-sized dorsal tubercles (12-16 longitudinal rows at mid-body); (6) large pointed tubercles on occiput, neck and sides of head; (7) keeled tubercles present on forearms and hind limbs but absent on upper arms; (8) paired terminal scansors on digits extending well beyond claws; (9) cloacal tubercles small; (10) subcaudal series of expanded scales reaching vent area anteriorly; (11) tail tip laterally compressed and feebly vertically expanded (Fig. 4C); (12) dorsum with a pattern of approximately 5 orange-brown transverse bars (one on neck, three on body and one on sacrum; Fig. 4C); (13) tail colour not sexually dimorphic; (14) adults with whitish-ivory tails (whiter distally) with 5–7 wide orange-dark crossbands (last 2–3 crossbands black and extending ventrally) (Fig. 4C); (15) juveniles with very conspicuous orange-coppery tails with 6–8 dark-brown crossbands not extending ventrally (Fig. 5A); (16) tail can be coiled and waved.

*Differential diagnosis*. *Asaccus margaritae* **sp. nov.** differs from *A. caudivolvulus* mainly in its smaller size (SVL max. 58.7 mm, compared with max. 63.3 mm), in having shorter fore limbs and hind limbs with respect to SVL (ANOVA comparisons of LUn, LHu and LTb all significant, *P* < 0.001, Table S4), in the size of the tubercles on the occiput, neck and sides of head (large size tubercles *versus* small pointed tubercles), in the absence of tubercles on the upper arm (tubercles on upper arm present on *A. caudivolvulus*), in the different tail colour in juveniles (orange-coppery tail *versus* white tail; Fig. 5). It further differs by a genetic distance of 26.3% and 16.2% in the mitochondrial *cytb* and *12S* genes, respectively (Table 2) and in the presence of different alleles in the *acm4*, *cmos* and *mc1r* nuclear genes analysed here (Fig. 2). For differences between *Asaccus margaritae* **sp. nov.** and *Asaccus gardneri* **sp. nov.** see “*differential diagnosis*” of *Asaccus gardneri* **sp. nov.** above. It differs from *Asaccus andersoni* in its smaller size (SVL max. 58.7 mm, compared with max. 66.2 mm), in the number and disposition of the postmentals (2 pairs of postmentals, the first pair in contact *versus* 2–3 pairs of postmentals, first pair separated by mental), in the presence of crossbands on the tail that extend ventrally (crossbands on tail do not extend ventrally in *A. andersoni*), in the size and shape of the tubercles on the dorsal body (keeled trihedral moderate-sized dorsal tubercles *versus* smooth tubercles), in the absence of sexual dichromatism (presence in *A. andersoni*). It differs from *Asaccus barani* in the paired terminal scansors on the digits (extend well beyond claws *versus* do not extend beyond claws) and in the tubercles on the upper arm (absent *versus* present). It differs from *Asaccus elisae* in the scales across the supraorbital region (fine *versus* coarse), in the absence of tubercles on the upper arm (tubercles on upper arm present on *A. elisae*), in the paired terminal scansors on the digits (extend well beyond claws *versus* do not extend beyond claws), in the ability to coil the tail laterally like a loose watch-spring (inability to curl the tail in *A. elisae*) and by a genetic distance of 20.9% in the mitochondrial *12S* gene,. It differs from *Asaccus gallagheri* in its larger size (SVL max. 58.7 mm, compared with max. 40 mm), in the presence of dorsal tubercles on the back, occiput, and elsewhere (absence of tubercles in *A. gallagheri*), in the tail tip laterally compressed and vertically expanded (tail tip not laterally compressed or vertically expanded in *A. gallagheri*), in the tail colour not being sexually dimorphic (tail colour sexually dimorphic in *A. gallagheri*; white with black crossbars in females and yellow in males), in the ability to coil the tail laterally like a loose watch-spring (inability to curl the tail in *A. gallagheri*) and by a genetic distance of 25.6% and 15.7% in the mitochondrial *cytb* and *12S* genes, respectively. It differs from *Asaccus granularis* in its smaller size (SVL max. 58.7 mm, compared with max. 70.2 mm), in the presence of tubercles on the occiput, neck and sides of head (absence in *A. granularis*), in the presence of 2–3 crossbands on the tail extending ventrally (crossbands on tail do not extend ventrally in *A. granularis*), in the ability to coil the tail laterally like a loose watch-spring (inability to curl the tail in *A. granularis*). It differs from *Asaccus griseonotus* in its smaller size (SVL max. 58.7 mm, compared with max. 71 mm), in the scales across the supraorbital region (fine *versus* coarse), in the dorsal tubercles (keeled trihedral moderate-sized dorsal tubercles present on back and large pointed tubercles on occiput, neck and sides of head *versus* small dorsal tubercles present on back but absent on occiput), in the presence of 2–3 crossbands on the tail extending ventrally (crossbands on tail do not extend ventrally in *A. griseonotus*), in the ability to coil the tail laterally like a loose watch-spring (inability to curl the tail in *A. griseonotus*) and by a genetic distance of 23.8% and 21.3% in the mitochondrial *cytb* and *12S* genes, respectively. It differs from *Asaccus iranicus* in its larger size (SVL max. 58.7 mm, compared with max. 41.8 mm), in the fore limb fingers not being parallel to the arm (parallel to the arm in *A. iranicus*; Figs. 3–4 of Torki et al., 2011a), in the paired terminal scansors on digits (extend well beyond claws *versus* do not extend beyond claws), in the absence of tubercles on the upper arm (tubercles on upper arm present on *A. iranicus*). It differs from *Asaccus kermanshahensis* in the number of postmentals (2 pairs *versus* 4 pairs), in the dorsal tubercles (keeled trihedral moderate-sized dorsal tubercles present on back and large pointed tubercles on occiput, neck and sides of head *versus* smooth oval or round dorsal tubercles present on occiput, neck and sides of head), in the presence of 2–3 crossbands on the tail extending ventrally (tail without dark rings in *A. kermanshahensis*). It differs from *Asaccus kurdistanensis* in the number of postmentals (2 pairs *versus* 3 pairs), in the dorsum colouration (dorsum pale pink with a pattern of approximately 5 orange-brown transverse bars -one on neck, three on body and one on sacrum- *versus* dorsum whitish-grey with dark large spots scattered throughout). It differs from *Asaccus montanus* in its larger size (SVL max. 58.7 mm, compared with max. 40 mm), in the dorsal tubercles (keeled trihedral moderate-sized dorsal tubercles present on back and large pointed tubercles on occiput, neck and sides of head *versus* very large keeled dorsal tubercles on back -some in contact-, occiput and sides of head), in the absence of tubercles on the upper arm (very large keeled tubercles on upper arm present in *A. montanus*), in the paired terminal scansors on the digits (extend well beyond claws *versus* do not extend beyond claws) and by a genetic distance of 27.5% and 21.5% in the mitochondrial *cytb* and *12S* genes, respectively. It differs from *Asaccus nasrullahi* in its smaller size (SVL max. 58.7 mm, compared with max. 70 mm), in the scales across the supraorbital region (fine *versus* coarse), in the dorsal tubercles (keeled trihedral moderate-sized dorsal tubercles present on back in 11–16 longitudinal rows at mid-body and large pointed tubercles on occiput, neck and sides of head *versus* small, circular tubercles on back in fewer than 9 longitudinal rows at mid-body and absence of tubercles on occiput and sides of head) and by a genetic distance of 25.8% and 17.7% in the mitochondrial *cytb* and *12S* genes, respectively. It differs from *Asaccus platyrhynchus* in the dorsal tubercles (keeled trihedral moderate-sized dorsal tubercles present on back *versus* small tubercles on back), in the tail tip laterally compressed and vertically expanded and tail colour not sexually dimorphic (tail tip not laterally compressed or vertically expanded and tail colour sexually dimorphic, white with black crossbars in females and yellow in males in *A. platyrhynchus*) and by a genetic distance of 26.2% and 13.7% in the mitochondrial *cytb* and *12S* genes, respectively. It differs from *Asaccus saffinae* in the dorsal tubercles (keeled trihedral moderate-sized dorsal tubercles present on back and large pointed tubercles on the occiput, neck and sides of head *versus* smooth, unkeeled dorsal tubercles present on back, a few very small tubercles on occiput and no tubercles on head), first pair of postmentals in contact (not in contact in *A. saffinae*). It differs from *Asaccus tangestanensis* in the body shape and limb length (robust body with relatively short extremities *versus* thin body with very elongated limbs), in the absence of tubercles on the upper arm (tubercles on upper arm present on *A. tangestanensis*), in the paired terminal scansors on the digits (extend well beyond claws *versus* do not extend beyond claws). It differs from *Asaccus zagrosicus* in the absence of tubercles on the upper arm (tubercles on upper arm present on *A. zagrosicus*), in the paired terminal scansors on the digits (extend well beyond claws *versus* do not extend beyond claws), in the secondary postmentals (in contact with lower labials *versus* separated from lower labials by 1–3 rows of scales).

*Description of the holotype.* BMNH2008.989. (Fig. 4C). Complete specimen with the tip of the tongue missing (used for DNA extraction). Data on 13 morphometric and five meristic variables (see Material and Methods) are provided in Table S2. Adult male, SVL 54.9 mm, depressed head and body, well-marked neck, relatively short limbs, snout strongly depressed with a concave upper profile in lateral view, head length 29% of SVL and head width 71% of head length, tail not regenerated 1.17 times SVL, rostral scale rectangular with upper edge with shallow W shape, more than twice as wide as high and without a media cleft, nostril bordered by rostral, first labial, internasal and two postnasal scales, all entering into its border, the two internasal scales in contact behind the rostral, three distinct depressions exist, one right behind the nostrils, another one right in front of the lower part of the eyes and the last one in an area comprised between the eyes down to the nostrils, snout area with flat scales and no tubercles, eyes large, diameter about 27% of head length, vertical pupil, palpebral fold edged anteriorly with enlarged imbricated scales that decrease in size towards the back and become ciliated, one row of supraorbital rounded tubercles, ear opening vertical (elliptical), smooth edged, approximately twice longer than wide, mental scale large, wedge-shaped, extending backwards to level of sutures between first and second lower labial scales, two pairs of postmental scales, the first pair in contact with first and second lower labials and larger than the second pair, which is only in contact with the second lower labials, first pair of postmentals in contact and extending backwards to the third lower labial scale, well before the suture between the third and fourth lower labials, gular scales flat and small, without tubercles, dorsum covered with uniform, flattened granules with rounded outline, interspersed with moderate-sized keeled trihedral tubercles, large pointed tubercles on occiput, neck and sides of head, keeled tubercles present on forearms and hind limbs but absent on upper arms, ventral scales flat, slightly imbricate and about twice the diameter of dorsal scales at mid-belly, larger in the pelvic region, underside of digits with enlarged scales (9 under the 4th digit) that become replaced by irregular transverse rows of smaller scales (three under the 4th digit), paired terminal scansors on digits longer than wide, projecting well beyond claws, tail distinctly dorsoventrally flattened at base, but round distally, tail tip laterally compressed and feebly vertically expanded, tail divided in segments covered above by small slightly imbricate scales and six large keeled trihedral tubercles on the basal segments, reduced to four smaller ones distally, underside of tail with a single series of expanded approximately rectangular scales, two per segment.

Colouration in alcohol whitish-yellow underneath and brownish above. Tail with six dark transverse crossbands increasing in intensity distally; ventral surface of tail pale with the three distal most crossbands extending into it very faintly. Colour in life much richer than in the fixed specimen (Fig. 4C), pale pink with five irregular transverse orange crossbands across the back (one on neck, three on body and one on sacrum). Several orange stripes present on the head and snout area, the first three tail crossbands orange and the three distal most tail crossbands black. Iris in life colourful, golden with dark venations.

*Variation.* Data on 13 morphometric and five meristic variables (see Material and Methods) for all 9 paratypes (see above) are provided in Table S2. Specimens IBECN10420-21 with regenerated tails and specimens IBECN10419 and IBECN10422 with complete tails, all four paratypes with an incision on the ventral side of the thigh from where muscular tissue was removed for DNA extraction. Specimen BMNH2008.988 with complete tail, IBECN8708, IBECN9023 and IBECN9012 with regenerated tails and ONHM4222 with broken tail, in all five specimens the tip of the tongue was cut and used for DNA extraction. All the specimens are very similar to each other, main colouration very similar to the holotype.

*Distribution and ecology.* Despite intensive sampling across the Hajar Mountain range and other areas in Arabia carried out between 2004 and 2016, *Asaccus margaritae* **sp. nov.** has only been found in a very few high altitude localities (1,315–1,434 m a.s.l.) in the Musandam Peninsula, Oman and in two other localities 75 and 120 km further South in Oman and the UAE, respectively, both at relatively low elevation (122 and 374 m a.s.l.) (Fig. 1A; Table S1). Like *Asaccus gardneri* **sp. nov.** it is an endemic species to the northernmost part of the Hajar Mountain range. *Asaccus margaritae* **sp. nov.** has been found on rocky sides of wadis, stony substrates and on larger boulders in the wadi. Strictly nocturnal, all specimens were captured during the night and avoided the beam of the flashlight, running into crevices, holes and fleeing across rocks with ease but with much less agility and speed than both *Asaccus gardneri* **sp. nov.** and *Asaccus caudivolvulus*, both larger, with relatively thinner bodies and longer limbs, adapted for high speed climbing, jumping and running. In comparison to *Asaccus gardneri* **sp. nov.** (very common over a small area in some localities like for instance Khasab; Arnold & Gardner, 1994; S Carranza, pers. obs., 2013), *A. margaritae* **sp. nov.** is very scarce everywhere and a maximum of 7 specimens have ever been found in a single night.

*Conservation status.* Not evaluated.

Proposal of common names:

English: Margarita’s Leaf-toed Gecko

Arabic: وزغةمارغريتاورقيةالأصابع

## Discussion

Taking an integrative approach, we have uncovered a very old diversification event that has resulted in a case of microendemicity in an arid mountain range. Three morphologically and ecologically similar medium sized lizard species were shown to coexist in a very short and narrow mountain stretch of the northern tip of the Hajar Mountain range of approximately 140 km from north to south and 40 km from east to west (4,350 km^2^). As suggested by Arnold & Gardner (1994), using morphological data and Papenfuss et al. (2010) using molecular data, the diversity of Arabian *Asaccus* is much higher than was previously thought and the two new species described herein increases the number of endemic reptile species of the Hajar Mountains to 19 (De Pous et al., 2016). Moreover, our study highlights a high level of diversity and microendemicity in the northern Hajar Mountains, exemplified by the presence of *Asaccus caudivolvulus*, which is currently the only vertebrate species known to be endemic to the UAE and is restricted to a very small coastal area of approximately 20 km (Fig. 1A). The discovery of this radiation in the northern tip of the Hajar Mountains highlights the importance of this area, especially given that it has previously received little scientific attention in contrast to areas further south in the Central and Eastern Hajar Mountains such as the Jebel Akhdar (Harrison, 1976; Harrison, 1977). While genetic studies carried out on the geckos of the genera *Hemidactylus* (Carranza & Arnold, 2012), *Pristurus* (Badiane et al., 2014; Carranza & M Simó-Riudalbas, pers. obs., 2015) and *Trachydactylus* (De Pous et al., 2016) indicate that the areas with the maximum genetic diversity are situated around the Central and Eastern Hajar Mountains, our current study of *Asaccus* and the results of previous studies on the genus *Ptyodactylus* (Metallinou et al., 2015) stress the need for a much more thorough investigation of the diversity of the Hajar Mountains in the UAE and the Musandam Peninsula. In fact, the Khor Fakkan area in the UAE (around locality 31 in Fig. 1A) is one of the narrowest and lowest mountain stretches of the Hajar Mountain range and is the only known area in which *Asaccus caudivolvulus*, *A. margaritae* **sp. nov.** and *A. gardneri* **sp. nov.** have been shown to occur in close proximity. Likewise, this area is also the location in which the discontinuity of the two deep genetic lineages of *Ptyodactylus orlovi* was identified (Metallinou et al., 2015). Importantly, the only area where the sole endemic vertebrate of the UAE (*Asaccus caudivolvulus*) lives is under heavy development. In particular, one of only two localities where *Asaccus caudivolvulus* has ever been found (locality 29 in Fig. 1A), is under heavy transformation and is zoned for dynamite blasting as part of the construction of a new tourist resort (Fig. 6), similar to developments already conducted or underway in other coastal areas of the Hajar Mountains. It is therefore of the utmost urgency that efforts are made to locate and protect other remaining populations of *A. caudivolvulus*, a species that has resided in this part of Arabia for approximately 4 Ma, long before human settlement (Stringer, 2011; Lordkipanidze et al., 2013). Concurrently, alternative *ex-situ* initiatives as applied to other vertebrates in similar situations, such as the establishment of a captive breeding programme in the range country offers a temporary solution until a suitable protected area can be established for this unique endemism.

Our findings demonstrate the utility of applying molecular data to uncover hidden diversity, something that has already been done with unprecedented results for the complete reptile fauna of the Socotra Archipelago in a pioneering DNA Barcode study of Arabian fauna (Vasconcelos et al., 2016). This kind of study, combining carefully planned explorations using available Geographical Information System (GIS) tools with relatively quick standardized molecular analyses should be applied to other biodiversity-rich areas of Arabia in order to investigate the existence of additional cases of hidden diversity susceptible (see, for examples Smid et al.,; Busais & Joger, 2011a; Busais & Joger, 2011b; Carranza & Arnold, 2012; Metallinou & Carranza, 2013; Šmíd et al., 2013; Šmíd et al., 2015, Šmíd et al., 2016; Badiane et al., 2014; Vasconcelos & Carranza, 2014; De Pous et al., 2016).

Similar to findings in a related species, *Asaccus gallagheri* (Papenfuss et al., 2010), our results reveal *A. montanus* to be the sister taxon to all the remaining *Asaccus* included in our study, demonstrating the Arabian *Asaccus* to be polyphyletic. Although this topology (Fig. 1B), together with the demonstrated sister taxon relationship between *Asaccus* and the endemic Arabian (Socotra Archipelago) gecko genus *Haemodracon* (Gamble et al., 2008; Gamble et al., 2011; Gamble et al., 2012; Garcia-Porta et al., 2016a; Garcia-Porta et al., 2016b) supports an Arabian origin for the genus *Asaccus*, the origin cannot be confirmed until the remaining species of the genus are included in a phylogenetic analysis. Nevertheless, the present study highlights the diversity of this genus in the Hajar Mountains, similar to findings for another Hajar endemic with several cryptic lineages, *Asaccus gallagheri* (Papenfuss et al., 2010; work in progress) and warrants special conservation consideration for the area’s biodiversity.

##  Supplemental Information

10.7717/peerj.2371/supp-1 Table S1 Information on the specimens used in the phylogenetic (sample code) and morphological (morphology = yes) analyses, with locality data and GenBank accession numbers Voucher codes of specimens available refer to the following collections; BMNH: British Museum of Natural History; CAS: California Academy of Sciences, USA; IBE: Institute of Evolutionary Biology (CSIC-UPF), Barcelona, Spain; MVZ: Museum of Vertebrate Zoology, California, Berkeley; ONHM: Oman Natural History Museum; SQU: Sultan Qaboos University, Oman. The holotype (*) and paratypes are underlined.

10.7717/peerj.2371/supp-2 Table S2 Sex, metric, meristic and categorical variables of all examined specimens of *A. gardneri* sp. nov. (1), *A. caudivolvulus* (2) and *A. margaritae* sp. nov. (3). Summary statistics for each species are also given. The holotype (*) and paratypes are underlined. SVL, Snout-vent length; TrL, Trunk lenght; HL, Head length; HW, Head width; HH, Head height; SL, Snout length; SW, Snou width; ED, Eye diameter; EVD, Ear vertical diameter; LUn, Ulna length; LHu, Humerus length; LTb, Tibia length; LFe, Femur length; Trow, Dorsal tubercles rows; LT4, Lamella rows under the 4th toe; ST4, Rows of enlarged distal scales under the 4th toe; ULS, Upper labial scales; LLS, Lower labial scales; TUA, Presence of tubercles on upper arm. Museum acronyms as in Table S1.

10.7717/peerj.2371/supp-3 Table S3 Loadings, eigenvalues, proportion and cumulative proportion of variance explained of the first six components selected from the PCA performed on shape residuals for each morphological variable The asterisk (*) signalling the component indicates that is significantly different between species and its loadings in bold indicates the most important variables. Abbreviations of each morphological variable as in Table S2 and in the material and methods.

10.7717/peerj.2371/supp-4 Table S4 Results of the one-way ANOVAs performed on each of the morphometric and meristic variables for the three pairs of species of the *Asaccus caudivolvulus* radiation compared in this study (A) *A. margaritae* **sp. nov.** *versus A. gardneri* **sp. nov.**; (B) *A. margaritae* **sp. nov.** *versus A. caudivolvulus* and (C) *A. gardneri* **sp. nov.** *versus A. caudivolvulus*. *P*-values in bold indicate high level of statistical significance. Abbreviations of each morphological variable as in Table S2 and in the material and methods.

10.7717/peerj.2371/supp-5 Figure S1 ML phylogenetic tree of 9 *Asaccus* species. The phylogeny is based on the concatenated sequences of two mitochondrial (*12S* and *cytb*) and three nuclear (*c-mos*, *MC1R* and *ACM4*) genes. Bootstrap values ≥70% of the ML analysis are shown next to the nodes.

10.7717/peerj.2371/supp-6 Figure S2 ML phylogenetic analyses of the three nuclear genes independently (A) *c-mos*, (B) *MC1R*, (C) *ACM4*. The dataset used were phased in order to show the two alleles of each specimen. All the haplotypes of the three nuclear genes are private for all *Asaccus* species, with the only exception of *Asaccus caudivolvulus* and *Asaccus gardneri* **sp. nov.**, which share one haplotype in the *CMOS* gene and one haplotype in the *MC1R* gene (see Fig. 2).

## Additional Information and Declarations

### Field Study Permissions

The following information was supplied relating to field study approvals (i.e., approving body and any reference numbers): All the necessary collecting and export permits for this study in Oman were issued by the Nature Conservation Department of the Ministry of Environment and Climate Affairs, Oman (Refs: 08/2005; 16/2008; 38/2010; 12/2011; 13/2013; 21/2013) and the research in the United Arab Emirates was done under the supervision and permission of the Environment and Protected Areas Authority, Government of Sharjah.

### DNA Deposition

The following information was supplied regarding the deposition of DNA sequences: All sequences have been deposited in GenBank database. Accession numbers for the DNA sequences included in this study are provided in Table S1.

### Data Availability

The following information was supplied regarding data availability: All molecular or morphological data related to this manuscript is available in Tables S1 and S2.

### New Species Registration

The following information was supplied regarding the registration of a newly described species: Asaccus gardneri urn:lsid:zoobank.org:act:0532F92E-5136-4E0C-A752-B4973590308D *Asaccus margaritae* urn:lsid:zoobank.org:act:F6966A21-28A1-4A36-92E9-B56833144C29 Publication LSID: urn:lsid:zoobank.org:pub:33DF71FE-3E0C-4907-8582-F7B369A0A713.
