# Supplementary material for: Microendemicity in the northern Hajar Mountains of Oman and the United Arab Emirates with the description of two new species of geckos of the genus Asaccus (Squamata: Phyllodactylidae)
Source: PeerJ. 2016 Aug 18;4:e2371. doi: 10.7717/peerj.2371 (PMC4994081; doi:10.7717/peerj.2371)
Supplement: Table S1 — Voucher codes of specimens available refer to the following collections; BMNH: British Museum of Natural History; CAS: California Academy of Sciences, USA; IBE: Institute of Evolutionary Biology (CSIC-UPF), Barcelona, Spain; MVZ: Museum of Vertebrate Zoology, California, Berkeley; ONHM: Oman Natural History Museum; SQU: Sultan Qaboos University, Oman. The holotype (*) and paratypes are underlined. [file peerj-04-2371-s001.docx]

| **Ingroup species** | **Sample code** | **Voucher code** | **Country** | **Elevation (m)** | **Locality** | **Morphology** | **12S** | **cytb** | ***ACM4*** | ***c-mos*** | ***MC1R*** |
| --- | --- | --- | --- | --- | --- | --- | --- | --- | --- | --- | --- |
| *A. gardneri* **sp. nov.** | CN2702 |  | Oman | 17 | 4 |  | KX550444 | KX550540 | KX550628 | KX550714 | KX550801 |
| *A. gardneri* **sp. nov.** | CN2755 | ONHM4221 | Oman | 17 | 4 | yes | KX550445 | KX550541 | KX550629 | KX550715 | KX550802 |
| *A. gardneri* **sp. nov.** | CN3042 | IBECN3042 | Oman | 17 | 4 | yes | KX550446 | KX550542 | KX550630 | KX550716 | KX550803 |
| *A. gardneri* **sp. nov.** | CN3462 |  | Oman | 4 | 3 |  | KX550479 | KX550572 | KX550659 | KX550746 | KX550832 |
| *A. gardneri* **sp. nov.** | CN3681 | IBECN3681 | Oman | 211 | 12 |  | KX550447 | KX550543 | KX550631 | KX550717 | KX550804 |
| *A. gardneri* **sp. nov.** | CN3686 |  | Oman | 4 | 3 |  | KX550448 | KX550544 | KX550632 | KX550718 | KX550805 |
| *A. gardneri* **sp. nov.** | CN3901 | IBECN3901 | Oman | 643 | 15 | yes | KX550487 | KX550580 | KX550667 | KX550754 | KX550840 |
| *A. gardneri* **sp. nov.** | CN3903 | IBECN3903 | Oman | 7 | 6 | yes | KX550480 | KX550573 | KX550660 | KX550747 | KX550833 |
| *A. gardneri* **sp. nov.** | CN3904 | IBECN3904 | Oman | 977 | 18 | yes | KX550463 | KX550558 | KX550646 | KX550732 | KX550819 |
| *A. gardneri* **sp. nov.** | CN3905 | BMNH2008.1000* | Oman | 16 | 10 | yes | KX550485 | KX550578 | KX550665 | KX550752 | KX550838 |
| *A. gardneri* **sp. nov.** | CN3907 | IBECN3907 | Oman | 7 | 6 | yes | KX550449 | KX550545 | KX550633 | KX550719 | KX550806 |
| *A. gardneri* **sp. nov.** | CN3910 | IBECN3910 | Oman | 7 | 6 | yes | KX550481 | KX550574 | KX550661 | KX550748 | KX550834 |
| *A. gardneri* **sp. nov.** | CN3914 | IBECN3914 | Oman | 7 | 6 | yes | KX550450 | KX550546 | KX550634 | KX550720 | KX550807 |
| *A. gardneri* **sp. nov.** | CN3955 | IBECN3955 | Oman | 280 | 25 | yes | KX550464 | KX550559 | KX550647 | KX550733 | KX550820 |
| *A. gardneri* **sp. nov.** | CN3965 | IBECN3965 | Oman | 6 | 11 | yes | KX550489 | KX550582 | KX550669 | KX550756 | KX550842 |
| *A. gardneri* **sp. nov.** | CN5771 | BMNH2008.999 | Oman | 17 | 4 | yes | KX550451 | KX550547 | KX550635 | KX550721 | KX550808 |
| *A. gardneri* **sp. nov.** | CN7264 | IBECN7264 | Oman | 6 | 5 |  | KX550460 | - | - | - | - |
| *A. gardneri* **sp. nov.** | CN751 | IBECN751 | Oman | 5 | 2 | yes | KX550452 | KX550548 | KX550636 | KX550722 | KX550809 |
| *A. gardneri* **sp. nov.** | CN757 | IBECN757 | Oman | 1 | 1 | yes | KX550453 | KX550549 | KX550637 | KX550723 | KX550810 |
| *A. gardneri* **sp. nov.** | CN760 | IBECN760 | Oman | 923 | 19 | yes | - | KX550618 | KX550704 | KX550791 | KX550877 |
| *A. gardneri* **sp. nov.** | CN763 |  | Oman | 643 | 15 |  | KX550454 | KX550550 | KX550638 | KX550724 | KX550811 |
| *A. gardneri* **sp. nov.** | CN784 | IBECN784 | Oman | 647 | 16 | yes | KX550482 | KX550575 | KX550662 | KX550749 | KX550835 |
| *A. gardneri* **sp. nov.** | CN797 | IBECN797 | Oman | 923 | 19 | yes | KX550486 | KX550579 | KX550666 | KX550753 | KX550839 |
| *A. gardneri* **sp. nov.** | CN801 | IBECN801 | Oman | 977 | 18 | yes | KX550455 | KX550551 | KX550639 | KX550725 | KX550812 |
| *A. gardneri* **sp. nov.** | CN8109 | IBECN8109 | Oman | 116 | 26 | yes | KX550469 | KX550562 | KX550649 | KX550736 | KX550823 |
| *A. gardneri* **sp. nov.** | CN817 | IBECN817 | Oman | 923 | 19 | yes | KX550456 | KX550552 | KX550640 | KX550726 | KX550813 |
| *A. gardneri* **sp. nov.** | CN8370 | IBECN8370 | Oman | 116 | 26 | yes | KX550472 | KX550565 | KX550652 | KX550739 | KX550826 |
| *A. gardneri* **sp. nov.** | CN842 | IBECN842 | Oman | 647 | 16 | yes | KX550488 | KX550581 | KX550668 | KX550755 | KX550841 |
| *A. gardneri* **sp. nov.** | CN844 | IBECN844 | Oman | 5 | 2 | yes | KX550457 | KX550553 | KX550641 | KX550727 | KX550814 |
| *A. gardneri* **sp. nov.** | CN848 | IBECN848 | Oman | 688 | 14 | yes | KX550458 | KX550554 | KX550642 | KX550728 | KX550815 |
| *A. gardneri* **sp. nov.** | CN8673 | IBECN8673 | Oman | 4 | 27 | yes | KX550470 | KX550563 | KX550650 | KX550737 | KX550824 |
| *A. gardneri* **sp. nov.** | CN8674 | IBECN8674 | Oman | 116 | 26 | yes | - | KX550619 | KX550705 | KX550792 | KX550878 |
| *A. gardneri* **sp. nov.** | CN8700 | IBECN8700 | UAE | 157 | 21 | yes | KX550477 | KX550570 | KX550657 | KX550744 | KX550830 |
| *A. gardneri* **sp. nov.** | CN8701 | IBECN8701 | Oman | 760 | 24 |  | KX550461 | KX550556 | KX550644 | KX550730 | KX550817 |
| *A. gardneri* **sp. nov.** | CN8715 | IBECN8715 | UAE | 157 | 21 | yes | KX550484 | KX550577 | KX550664 | KX550751 | KX550837 |
| *A. gardneri* **sp. nov.** | CN9008 | IBECN9008 | UAE | 280 | 30 | yes | KX550471 | KX550564 | KX550651 | KX550738 | KX550825 |
| *A. gardneri* **sp. nov.** | CN9009 |  | UAE | 256 | 32 |  | KX550478 | KX550571 | KX550658 | KX550745 | KX550831 |
| *A. gardneri* **sp. nov.** | CN9024 | IBECN9024 | UAE | 157 | 21 |  | KX550462 | KX550557 | KX550645 | KX550731 | KX550818 |
| *A. gardneri* **sp. nov.** | TW1020 | IBECN10423 | Oman | 280 | 25 | yes | KX550465 | - | - | - | KX550821 |
| *A. gardneri* **sp. nov.** | TW1021 | IBECN10426 | Oman | 280 | 25 | yes | KX550473 | KX550566 | KX550653 | KX550740 | KX550827 |
| *A. gardneri* **sp. nov.** | TW1022 | IBECN10427 | Oman | 280 | 25 | yes | KX550466 | KX550560 | KX550648 | KX550734 | KX550822 |
| *A. gardneri* **sp. nov.** | TW1023 | IBECN10424 | Oman | 280 | 25 | yes | KX550467 | KX550561 | - | KX550735 | - |
| *A. gardneri* **sp. nov.** | TW1024 |  | Oman | 280 | 25 |  | KX550468 | - | - | - | - |
| *A. gardneri* **sp. nov.** | TW1025 |  | Oman | 280 | 25 |  | KX550474 | KX550567 | KX550654 | KX550741 | KX550828 |
| *A. gardneri* **sp. nov.** | TW1028 | IBECN10428 | UAE | 200 | 28 | yes | KX550476 | KX550569 | KX550656 | KX550743 | KX550829 |
| *A. gardneri* **sp. nov.** | TW1036 | IBECN10425 | Oman | 280 | 25 | yes | KX550475 | KX550568 | KX550655 | KX550742 | - |
| *A. gardneri* **sp. nov.** | UAE3 |  | Oman | 664 | 13 |  | KX550490 | KX550583 | KX550670 | KX550757 | KX550843 |
| *A. gardneri* **sp. nov.** | UAE4 |  | Oman | 921 | 17 |  | KX550483 | KX550576 | KX550663 | KX550750 | KX550836 |
| *A. gardneri* **sp. nov.** | UAE5 |  | Oman | 921 | 17 |  | KX550459 | KX550555 | KX550643 | KX550729 | KX550816 |
| *A. gardneri* **sp. nov.** |  | BMNH1976.1414 | Oman | 8 | 7 | yes |  |  |  |  |  |
| *A. gardneri* **sp. nov.** |  | BMNH1976.1415 | Oman | 8 | 7 | yes |  |  |  |  |  |
| *A. gardneri* **sp. nov.** |  | BMNH1976.1416 | Oman | 8 | 7 | yes |  |  |  |  |  |
| *A. gardneri* **sp. nov.** |  | BMNH1976.1417 | Oman | 8 | 7 | yes |  |  |  |  |  |
| *A. gardneri* **sp. nov.** |  | BMNH1976.1418 | Oman | 8 | 7 | yes |  |  |  |  |  |
| *A. gardneri* **sp. nov.** |  | BMNH1976.1419 | Oman | 8 | 7 | yes |  |  |  |  |  |
| *A. gardneri* **sp. nov.** |  | SQU1988.48 | Oman | 16 | 9 | yes |  |  |  |  |  |
| *A. gardneri* **sp. nov.** |  | SQU1988.49 | Oman | 31 | 8 | yes |  |  |  |  |  |
| *A. caudivolvulus* | S7445 | IBES7445 | UAE | 0 | 29 | yes | KX550491 | KX550584 | KX550671 | KX550758 | KX550844 |
| *A. caudivolvulus* | S7866 | IBES7866 | UAE | 0 | 29 | yes | KX550492 | KX550585 | KX550672 | KX550759 | KX550845 |
| *A. caudivolvulus* | S8088 | IBES8088 | UAE | 0 | 29 | yes | KX550493 | KX550586 | KX550673 | KX550760 | KX550846 |
| *A. caudivolvulus* | UAE26 |  | UAE | 0 | 29 |  | KX550494 | KX550587 | KX550674 | KX550761 | KX550847 |
| *A. caudivolvulus* | UAE27 |  | UAE | 0 | 29 |  | KX550495 | KX550588 | KX550675 | KX550762 | KX550848 |
| *A. caudivolvulus* |  | BMNH1973.1850* | UAE | 186 | 31 | yes |  |  |  |  |  |
| *A. caudivolvulus* |  | BMNH1973.18951 | UAE | 186 | 31 | yes |  |  |  |  |  |
| *A. margaritae* **sp. nov.** | CAS250891 | CAS250891 | Oman | 122 | 33 |  | KX550522 | KX550615 | KX550701 | KX550788 | KX550874 |
| *A. margaritae* **sp. nov.** | CAS250892 | CAS250891 | Oman | 122 | 33 |  | KX550524 | KX550617 | KX550703 | KX550790 | KX550876 |
| *A. margaritae* **sp. nov.** | CN2997 | IBECN2997 | Oman | 1315 | 20 | yes | KX550496 | KX550589 | KX550676 | KX550763 | KX550849 |
| *A. margaritae* **sp. nov.** | CN3419 | IBECN3419 | Oman | 1434 | 22 |  | KX550497 | KX550590 | KX550677 | KX550764 | KX550850 |
| *A. margaritae* **sp. nov.** | CN3592 | IBECN3592 | Oman | 1434 | 22 |  | KX550498 | KX550591 | KX550678 | KX550765 | KX550851 |
| *A. margaritae* **sp. nov.** | CN3908 | ONHM4222 | UAE | 373 | 34 | yes | KX550505 | KX550598 | KX550685 | KX550772 | KX550858 |
| *A. margaritae* **sp. nov.** | CN3966 | BMNH2008.989* | UAE | 373 | 34 | yes | KX550517 | KX550610 | KX550696 | KX550783 | KX550869 |
| *A. margaritae* **sp. nov.** | CN3967 | BMNH2008.988 | UAE | 373 | 34 | yes | KX550523 | KX550616 | KX550702 | KX550789 | KX550875 |
| *A. margaritae* **sp. nov.** | CN7126 | IBECN7126 | Oman | 1434 | 22 |  | KX550502 | KX550595 | KX550682 | KX550769 | KX550855 |
| *A. margaritae* **sp. nov.** | CN742 |  | UAE | 373 | 34 |  | KX550506 | KX550599 | KX550686 | KX550773 | KX550859 |
| *A. margaritae* **sp. nov.** | CN744 |  | UAE | 373 | 34 |  | KX550507 | KX550600 | KX550687 | KX550774 | KX550860 |
| *A. margaritae* **sp. nov.** | CN749 |  | UAE | 373 | 34 |  | KX550508 | KX550601 | KX550688 | KX550775 | KX550861 |
| *A. margaritae* **sp. nov.** | CN8191 | IBECN8191 | Oman | 1434 | 22 | yes | KX550499 | KX550592 | KX550679 | KX550766 | KX550852 |
| *A. margaritae* **sp. nov.** | CN8195 | IBECN8195 | Oman | 1434 | 22 |  | KX550500 | KX550593 | KX550680 | KX550767 | KX550853 |
| *A. margaritae* **sp. nov.** | CN840 |  | UAE | 373 | 34 |  | KX550509 | KX550602 | KX550689 | KX550776 | KX550862 |
| *A. margaritae* **sp. nov.** | CN847 |  | UAE | 373 | 34 |  | KX550503 | KX550596 | KX550683 | KX550770 | KX550856 |
| *A. margaritae* **sp. nov.** | CN861 |  | UAE | 373 | 34 |  | KX550510 | KX550603 | KX550690 | KX550777 | KX550863 |
| *A. margaritae* **sp. nov.** | CN8631 | IBECN8631 | Oman | 1349 | 23 |  | KX550501 | KX550594 | KX550681 | KX550768 | KX550854 |
| *A. margaritae* **sp. nov.** | CN864 |  | UAE | 346 | 36 |  | KX550511 | KX550604 | KX550691 | KX550778 | KX550864 |
| *A. margaritae* **sp. nov.** | CN865 |  | UAE | 373 | 34 |  | KX550512 | KX550605 | KX550692 | KX550779 | KX550865 |
| *A. margaritae* **sp. nov.** | CN8708 | IBECN8708 | UAE | 373 | 34 | yes | KX550518 | KX550611 | KX550697 | KX550784 | KX550870 |
| *A. margaritae* **sp. nov.** | CN9012 | IBECN9012 | UAE | 373 | 34 | yes | KX550519 | KX550612 | KX550698 | KX550785 | KX550871 |
| *A. margaritae* **sp. nov.** | CN9020 | IBECN9020 | UAE | 373 | 34 | yes | KX550520 | KX550613 | KX550699 | KX550786 | KX550872 |
| *A. margaritae* **sp. nov.** | CN9023 | IBECN9023 | UAE | 373 | 34 | yes | KX550521 | KX550614 | KX550700 | KX550787 | KX550873 |
| *A. margaritae* **sp. nov.** | TW1017 | IBECN10419 | UAE | 374 | 35 | yes | KX550513 | KX550606 | KX550693 | KX550780 | KX550866 |
| *A. margaritae* **sp. nov.** | TW1031 | IBECN10420 | UAE | 374 | 35 | yes | KX550504 | KX550597 | KX550684 | KX550771 | KX550857 |
| *A. margaritae* **sp. nov.** | TW1032 | IBECN10421 | UAE | 374 | 35 | yes | KX550514 | KX550607 | KX550694 | KX550781 | KX550867 |
| *A. margaritae* **sp. nov.** | TW1033 | IBECN10422 | UAE | 374 | 35 | yes | KX550515 | KX550608 | - | - | - |
| *A. margaritae* **sp. nov.** | TW1034 |  | UAE | 374 | 35 |  | KX550516 | KX550609 | KX550695 | KX550782 | KX550868 |
| *A. platyrhynchus* | S1751 | IBES1751 | Oman |  |  |  | KX550525 | KX550533 | KX550620 | KX550706 | KX550793 |
| *A. gallagheri* | CN4310 | IBECN4310 | Oman |  |  |  | KX550526 | KX550534 | KX550621 | KX550707 | KX550794 |
| *A. griseonotus* | MVZ234326 | MVZ234326 | Iran |  |  |  | KX550528 | KX550535 | KX550623 | KX550709 | KX550796 |
| *A. elisae* | MVZ234315 | MVZ234315 | Iran |  |  |  | KX550527 | - | KX550622 | KX550708 | KX550795 |
| *A. nasrullahi* | MVZ234330 | MVZ234330 | Iran |  |  |  | KX550529 | KX550536 | KX550624 | KX550710 | KX550797 |
| *A. montanus* | AO43 |  | Oman |  |  |  | KX550530 | KX550537 | KX550625 | KX550711 | KX550798 |
|  |  |  |  |  |  |  |  |  |  |  |  |
| **Outgroup species** |  |  |  |  |  |  |  |  |  |  |  |
| *H. riebeckii* | S5011 |  | Yemen (Socotra) |  |  |  | KX550532 | KX550539 | KX550627 | KX550713 | KX550800 |
| *H. trachyrinus* | S5290 |  | Yemen (Socotra) |  |  |  | KX550531 | KX550538 | KX550626 | KX550712 | KX550799 |
