# Supplementary material for: Microendemicity in the northern Hajar Mountains of Oman and the United Arab Emirates with the description of two new species of geckos of the genus Asaccus (Squamata: Phyllodactylidae)
Source: PeerJ. 2016 Aug 18;4:e2371. doi: 10.7717/peerj.2371 (PMC4994081; doi:10.7717/peerj.2371)
Supplement: Table S2 — Summary statistics for each species are also given. The holotype (*) and paratypes are underlined. SVL, Snout-vent length; TrL, Trunk lenght; HL, Head length; HW, Head width; HH, Head height; SL, Snout length; SW, Snou width; ED, Eye diameter; EVD, Ear vertical diameter; LUn, Ulna length; LHu, Humerus length; LTb, Tibia length; LFe, Femur length; Trow, Dorsal tubercles rows; LT4, Lamella rows under the 4th toe; ST4, Rows of enlarged distal scales under the 4th toe; ULS, Upper labial scales; LLS, Lower labial scales; TUA, Presence of tubercles on upper arm. Museum acronyms as in Table S1. [file peerj-04-2371-s002.docx]

| **Species** | **Voucher code** | **Sex** | **SVL** | **TrL** | **HL** | **HW** | **HH** | **SL** | **SW** | **ED** | **EVD** | **LUn** | **LHu** | **LTb** | **LFe** | **Trow** | **LT4** | **ST4** | **ULS** | **LLS** | **TUA** |
| --- | --- | --- | --- | --- | --- | --- | --- | --- | --- | --- | --- | --- | --- | --- | --- | --- | --- | --- | --- | --- | --- |
| 1 | BMNH1976.1416 | M | 60.4 | 25.6 | 17.9 | 11.1 | 7.1 | 7.0 | 9.3 | 5.2 | 2.4 | 11.5 | 9.2 | 14.7 | 16.0 | 14 | 9 | 3 | 13 | 10 | 0 |
| 1 | BMNH1976.1417 | M | 59.6 | 25.0 | 18.4 | 10.7 | 7.1 | 7.3 | 9.0 | 5.1 | 2.3 | 11.4 | 9.1 | 14.6 | 15.7 | 14 | 9 | 3 | 14 | 10 | 0 |
| 1 | BMNH1976.1418 | M | 56.1 | 22.4 | 17.3 | 10.5 | 6.4 | 6.7 | 8.8 | 5.1 | 2.7 | 9.8 | 8.8 | 12.4 | 13.5 | 14 | 10 | 3 | 14 | 10 | 0 |
| 1 | BMNH2008.1000* | M | 63.4 | 29.3 | 18.3 | 13.2 | 8.8 | 7.0 | 9.5 | 4.7 | 2.7 | 11.6 | 9.1 | 14.0 | 14.7 | 15 | 10 | 4 | 14 | 10 | 0 |
| 1 | IBECN3907 | M | 66.8 | 26.1 | 19.2 | 13.3 | 9.1 | 7.8 | 10.8 | 5.1 | 3.1 | 13.0 | 10.0 | 15.9 | 15.4 | 15 | 11 | 3 | 15 | 11 | 0 |
| 1 | IBECN3910 | M | 60.9 | 25.0 | 17.9 | 11.1 | 6.6 | 8.8 | 10.1 | 4.5 | 2.5 | 11.8 | 9.5 | 14.0 | 15.6 | 13 | 10 | 4 | 14 | 11 | 0 |
| 1 | IBECN3955 | M | 68.4 | 28.2 | 20.0 | 13.5 | 8.8 | 9.3 | 11.0 | 4.8 | 2.9 | 13.7 | 9.7 | 15.8 | 17.1 | 14 | 9 | 3 | 15 | 12 | 0 |
| 1 | IBECN751 | M | 67.0 | 27.2 | 19.8 | 13.8 | 8.3 | 7.2 | 10.6 | 5.2 | 3.0 | 12.8 | 9.6 | 14.8 | 16.6 | 14 | 10 | 3 | 12 | 9 | 0 |
| 1 | IBECN757 | M | 66.8 | 27.0 | 19.6 | 12.0 | 7.5 | 9.2 | 10.3 | 5.4 | 2.1 | 11.6 | 9.2 | 14.9 | 15.3 | 14 | 10 | 4 | 12 | 9 | 0 |
| 1 | IBECN784 | M | 64.7 | 25.8 | 19.5 | 12.4 | 7.9 | 7.8 | 10.9 | 5.8 | 2.6 | 12.3 | 10.0 | 15.0 | 16.7 | 14 | 10 | 3 | 14 | 10 | 0 |
| 1 | IBECN797 | M | 68.6 | 28.3 | 19.9 | 14.1 | 8.4 | 7.1 | 10.3 | 5.5 | 2.6 | 13.7 | 9.7 | 16.1 | 16.8 | 12 | 11 | 4 | 14 | 11 | 0 |
| 1 | IBECN842 | M | 63.9 | 28.6 | 19.0 | 12.1 | 7.8 | 8.1 | 10.3 | 5.0 | 2.6 | 13.6 | 9.7 | 15.8 | 16.4 | 13 | 10 | 4 | 14 | 11 | 0 |
| 1 | IBECN848 | M | 69.8 | 28.1 | 20.9 | 12.6 | 8.0 | 9.1 | 10.9 | 5.8 | 3.2 | 12.9 | 11.0 | 16.1 | 16.5 | 14 | 9 | 3 | 16 | 11 | 0 |
| 1 | IBECN9008 | M | 59.0 | 23.7 | 17.7 | 10.8 | 6.4 | 7.4 | 9.6 | 5.1 | 2.8 | 10.9 | 9.6 | 13.8 | 14.0 | 14 | 9 | 3 | 14 | 10 | 0 |
| 1 | SQU1988.48 | M | 70.7 | 29.8 | 20.7 | 12.9 | 8.1 | 8.1 | 9.9 | 5.5 | 3.3 | 13.8 | 12.2 | 16.8 | 18.0 | 14 | 9 | 3 | 13 | 11 | 0 |
| 1 | IBECN10426 | M | 62.0 | 26.7 | 18.7 | 12.0 | 7.3 | 6.6 | 10.0 | 4.6 | 3.3 | 11.9 | 10.0 | 15.0 | 17.0 | 15 | 9 | 4 | 14 | 11 | 0 |
| 1 | IBECN10427 | M | 63.7 | 26.4 | 19.7 | 12.1 | 7.8 | 7.8 | 10.5 | 4.7 | 3.0 | 12.2 | 9.9 | 15.5 | 16.4 | 14 | 9 | 3 | 13 | 10 | 0 |
| 1 | BMNH1976.1414 | F | 62.0 | 24.2 | 18.4 | 11.9 | 6.9 | 7.6 | 9.9 | 5.5 | 2.8 | 11.1 | 9.2 | 14.7 | 15.5 | 14 | 9 | 3 | 14 | 9 | 0 |
| 1 | BMNH1976.1415 | F | 63.6 | 24.5 | 19.3 | 12.0 | 7.1 | 7.8 | 9.6 | 5.0 | 2.6 | 11.9 | 9.3 | 14.8 | 16.4 | 15 | 9 | 3 | 15 | 12 | 0 |
| 1 | BMNH1976.1419 | F | 54.0 | 20.9 | 16.6 | 10.1 | 6.4 | 6.6 | 8.3 | 4.6 | 2.5 | 10.3 | 8.5 | 13.8 | 14.1 | 15 | 10 | 3 | 14 | 10 | 0 |
| 1 | ONHM4221 | F | 64.0 | 26.5 | 19.0 | 11.7 | 7.6 | 8.2 | 10.3 | 5.1 | 2.7 | 12.0 | 9.1 | 14.6 | 16.5 | 14 | 10 | 3 | 14 | 10 | 0 |
| 1 | IBECN3042 | F | 62.9 | 27.0 | 19.0 | 11.7 | 7.1 | 8.1 | 10.0 | 5.0 | 2.6 | 11.5 | 9.5 | 14.0 | 14.9 | 14 | 9 | 3 | 13 | 9 | 0 |
| 1 | IBECN3901 | F | 57.9 | 22.7 | 17.4 | 10.7 | 6.4 | 7.1 | 9.1 | 5.4 | 2.0 | 11.2 | 9.7 | 14.9 | 15.2 | 14 | 10 | 3 | 15 | 10 | 0 |
| 1 | IBECN3903 | F | 51.8 | 19.8 | 16.1 | 10.2 | 6.3 | 6.4 | 8.3 | 5.0 | 1.9 | 10.1 | 8.4 | 12.9 | 12.6 | 14 | 10 | 3 | 13 | 10 | 0 |
| 1 | IBECN3904 | F | 70.7 | 30.0 | 20.6 | 13.4 | 9.0 | 9.0 | 11.4 | 5.2 | 3.0 | 14.3 | 11.1 | 17.4 | 17.8 | 15 | 10 | 3 | 14 | 11 | 0 |
| 1 | IBECN3914 | F | 61.5 | 24.9 | 18.4 | 11.8 | 7.6 | 7.7 | 9.8 | 4.9 | 2.4 | 11.4 | 9.5 | 13.9 | 14.2 | 14 | 9 | 3 | 12 | 9 | 0 |
| 1 | IBECN3965 | F | 60.7 | 25.1 | 18.3 | 11.4 | 8.0 | 7.4 | 9.8 | 5.0 | 2.6 | 11.9 | 9.7 | 14.5 | 16.3 | 13 | 9 | 3 | 14 | 10 | 0 |
| 1 | BMNH2008.999 | F | 61.6 | 26.0 | 18.1 | 11.2 | 7.4 | 7.6 | 10.1 | 5.0 | 2.3 | 12.3 | 8.6 | 13.9 | 15.5 | 14 | 9 | 3 | 15 | 10 | 0 |
| 1 | IBECN760 | F | 67.4 | 28.0 | 19.6 | 13.2 | 8.4 | 7.4 | 10.7 | 5.3 | 2.3 | 13.5 | 10.7 | 16.4 | 16.7 | 14 | 10 | 3 | 12 | 10 | 0 |
| 1 | IBECN801 | F | 69.2 | 30.0 | 21.0 | 13.7 | 7.4 | 8.9 | 11.4 | 5.0 | 3.2 | 13.4 | 9.7 | 16.2 | 16.8 | 14 | 10 | 3 | 13 | 11 | 0 |
| 1 | IBECN8109 | F | 56.8 | 21.1 | 17.4 | 9.6 | 6.9 | 7.3 | 8.3 | 4.6 | 2.1 | 10.4 | 8.7 | 14.2 | 13.8 | 11 | 9 | 3 | 14 | 11 | 0 |
| 1 | IBECN817 | F | 54.8 | 22.7 | 16.5 | 10.7 | 6.3 | 6.9 | 9.3 | 4.8 | 1.9 | 10.6 | 9.1 | 13.5 | 13.3 | 16 | 10 | 3 | 14 | 10 | 0 |
| 1 | IBECN8370 | F | 59.9 | 24.4 | 18.0 | 10.3 | 6.4 | 8.1 | 9.5 | 4.8 | 2.6 | 11.0 | 9.4 | 12.9 | 14.8 | 13 | 9 | 3 | 14 | 10 | 0 |
| 1 | IBECN844 | F | 61.5 | 25.6 | 18.7 | 12.6 | 7.5 | 7.6 | 9.8 | 5.2 | 2.5 | 12.0 | 9.0 | 13.5 | 16.1 | 13 | 9 | 3 | 13 | 9 | 0 |
| 1 | IBECN8673 | F | 59.5 | 22.5 | 18.0 | 12.0 | 8.2 | 7.8 | 9.9 | 4.5 | 2.7 | 12.2 | 9.7 | 14.4 | 14.5 | 13 | 9 | 3 | 14 | 11 | 0 |
| 1 | IBECN8674 | F | 57.6 | 21.5 | 17.5 | 11.1 | 6.2 | 7.3 | 9.5 | 4.8 | 2.5 | 10.8 | 10.1 | 13.4 | 15.0 | 14 | 11 | 3 | 15 | 11 | 0 |
| 1 | IBECN8700 | F | 52.8 | 20.8 | 15.7 | 9.9 | 5.8 | 6.7 | 8.7 | 4.6 | 2.4 | 10.9 | 9.3 | 13.2 | 13.5 | 12 | 10 | 3 | 14 | 10 | 0 |
| 1 | IBECN8715 | F | 65.4 | 29.5 | 19.0 | 12.1 | 8.5 | 7.6 | 10.0 | 5.1 | 2.2 | 12.2 | 10.4 | 15.8 | 16.4 | 15 | 11 | 3 | 15 | 11 | 0 |
| 1 | SQU1988.49 | F | 62.0 | 25.6 | 17.6 | 11.0 | 7.5 | 7.6 | 9.3 | 5.3 | 2.8 | 11.8 | 10.2 | 14.9 | 16.6 | 15 | 10 | 3 | 13 | 11 | 0 |
| 1 | IBECN10423 | F | 65.4 | 27.4 | 20.7 | 13.6 | 7.2 | 7.8 | 10.5 | 5.4 | 3.0 | 12.6 | 10.0 | 15.5 | 15.9 | 15 | 10 | 3 | 14 | 10 | 0 |
| 1 | IBECN10424 | F | 60.6 | 26.7 | 18.1 | 12.1 | 7.4 | 7.4 | 10.3 | 4.8 | 2.5 | 11.1 | 9.1 | 13.7 | 16.2 | 15 | 10 | 3 | 12 | 9 | 0 |
| 1 | IBECN10428 | F | 59.9 | 22.6 | 18.7 | 11.1 | 7.1 | 7.3 | 9.4 | 4.8 | 2.7 | 11.2 | 8.9 | 13.7 | 14.5 | 15 | 9 | 3 | 14 | 10 | 0 |
| 1 | IBECN10425 | F | 63.3 | 24.5 | 19.3 | 11.9 | 8.0 | 8.3 | 10.5 | 5.2 | 3.2 | 12.6 | 11.0 | 15.1 | 15.8 | 14 | 10 | 3 | 13 | 9 | 0 |
|  | **Summary Statistics (Total)** |  |  |  |  |  |  |  |  |  |  |  |  |  |  |  |  |  |  |  |  |
|  | Mean |  | 62.3 | 25.5 | 18.7 | 11.8 | 7.4 | 7.7 | 9.9 | 5.1 | 2.6 | 11.9 | 9.6 | 14.7 | 15.6 | 14 | 9.7 | 3.1 | 13.8 | 10.2 | - |
|  | Standart Deviation |  | 4.7 | 2.7 | 1.2 | 1.1 | 0.8 | 0.7 | 0.7 | 0.3 | 0.3 | 1.1 | 0.7 | 1.1 | 1.2 | 1 | 0.7 | 0.4 | 1 | 0.8 | - |
|  | Minimum |  | 51.8 | 19.8 | 15.7 | 9.6 | 5.8 | 6.4 | 8.3 | 4.5 | 1.9 | 9.8 | 8.4 | 12.4 | 12.6 | 11 | 9 | 3 | 12 | 9 | - |
|  | Maximum |  | 70.7 | 30.0 | 21.0 | 14.1 | 9.1 | 9.3 | 11.4 | 5.8 | 3.3 | 14.3 | 12.2 | 17.4 | 18.0 | 16 | 11 | 4 | 16 | 12 | - |
| 2 | BMNH1973.1850* | M | 63.2 | 26.5 | 18.7 | 13.0 | 7.3 | 7.2 | 10.1 | 5.0 | 3.2 | 10.0 | 9.2 | 14.0 | 15.7 | 15 | 10 | 3 | 13 | 11 | 1 |
| 2 | BMNH1973.18951 | M | 62.9 | 27.7 | 18.6 | 11.6 | 7.0 | 7.0 | 9.3 | 5.4 | 2.9 | 10.8 | 9.3 | 13.8 | 15.6 | 16 | 9 | 3 | 15 | 11 | 1 |
| 2 | IBES7445 | M | 61.3 | 25.7 | 18.9 | 12.0 | 7.4 | 7.7 | 10.3 | 4.8 | 2.7 | 10.8 | 8.9 | 12.5 | 14.1 | 14 | 9 | 3 | 14 | 9 | 1 |
| 2 | IBES8088 | M | 53.6 | 21.9 | 15.9 | 9.9 | 6.2 | 6.7 | 8.7 | 4.3 | 2.1 | 9.3 | 7.9 | 12.0 | 13.0 | 16 | 9 | 3 | 13 | 9 | 1 |
| 2 | IBES7866 | F | 58.5 | 23.9 | 17.7 | 11.6 | 6.4 | 8.2 | 9.9 | 4.9 | 2.1 | 10.0 | 8.7 | 12.3 | 13.1 | 16 | 8 | 4 | 13 | 9 | 1 |
|  | **Summary Statistics (Total)** |  |  |  |  |  |  |  |  |  |  |  |  |  |  |  |  |  |  |  |  |
|  | Mean |  | 59.9 | 25.1 | 18.0 | 11.6 | 6.9 | 7.4 | 9.7 | 4.9 | 2.6 | 10.2 | 8.8 | 12.9 | 14.3 | 15.4 | 9 | 3.2 | 13.6 | 9.8 | - |
|  | Standart Deviation |  | 4.0 | 2.2 | 1.2 | 1.1 | 0.5 | 0.5 | 0.6 | 0.3 | 0.4 | 0.6 | 0.5 | 0.9 | 1.3 | 0.9 | 0.7 | 0.5 | 0.9 | 1.1 | - |
|  | Minimum |  | 53.6 | 21.9 | 15.9 | 9.9 | 6.2 | 6.7 | 8.7 | 4.3 | 2.1 | 9.3 | 7.9 | 12.0 | 13.0 | 14 | 8 | 3 | 13 | 9 | - |
|  | Maximum |  | 63.2 | 27.7 | 18.9 | 13.0 | 7.4 | 8.2 | 10.3 | 5.4 | 3.2 | 10.8 | 9.3 | 14.0 | 15.7 | 16 | 10 | 4 | 15 | 11 | - |
| 3 | IBECN2997 | M | 58.6 | 26.1 | 17.1 | 11.8 | 6.2 | 7.7 | 9.5 | 3.6 | 2.3 | 9.3 | 7.7 | 11.2 | 12.5 | 14 | 9 | 3 | 13 | 10 | 0 |
| 3 | BMNH2008.989* | M | 54.8 | 23.4 | 15.8 | 11.2 | 6.2 | 7.0 | 8.9 | 4.2 | 1.9 | 8.9 | 7.8 | 10.6 | 12.2 | 14 | 9 | 3 | 14 | 10 | 0 |
| 3 | IBECN8191 | M | 51.7 | 21.6 | 15.2 | 10.5 | 4.8 | 6.3 | 8.7 | 3.8 | 1.3 | 8.1 | 6.7 | 10.2 | 10.5 | 13 | 8 | 3 | 14 | 10 | 0 |
| 3 | IBECN8708 | M | 56.0 | 24.5 | 16.2 | 10.5 | 5.9 | 6.9 | 9.0 | 4.2 | 2.0 | 8.5 | 7.9 | 10.8 | 12.0 | 13 | 7 | 3 | 13 | 10 | 0 |
| 3 | IBECN9012 | M | 53.7 | 23.4 | 15.5 | 10.1 | 6.1 | 6.9 | 9.0 | 3.9 | 2.0 | 8.9 | 7.6 | 10.5 | 11.5 | 16 | 8 | 3 | 14 | 11 | 0 |
| 3 | IBECN9020 | M | 53.8 | 22.5 | 15.9 | 10.6 | 6.1 | 6.6 | 8.8 | 4.1 | 2.1 | 8.5 | 7.2 | 10.4 | 12.0 | 13 | 8 | 3 | 13 | 10 | 0 |
| 3 | IBECN9023 | M | 56.3 | 22.9 | 15.9 | 10.2 | 5.6 | 7.1 | 8.9 | 4.4 | 1.9 | 8.6 | 8.2 | 10.4 | 12.0 | 12 | 8 | 2 | 14 | 10 | 0 |
| 3 | IBECN10419 | M | 53.9 | 18.9 | 15.9 | 11.5 | 6.8 | 6.4 | 8.8 | 3.8 | 2.0 | 8.3 | 7.2 | 10.3 | 12.8 | 16 | 8 | 3 | 13 | 9 | 0 |
| 3 | IBECN10420 | M | 55.7 | 22.6 | 16.7 | 11.7 | 7.6 | 7.2 | 9.4 | 3.9 | 2.0 | 8.9 | 7.2 | 10.9 | 12.0 | 14 | 8 | 3 | 13 | 10 | 0 |
| 3 | IBECN10421 | M | 52.9 | 22.3 | 16.4 | 11.4 | 7.0 | 6.5 | 9.0 | 4.0 | 1.7 | 8.0 | 7.3 | 9.0 | 13.1 | 15 | 8 | 3 | 12 | 9 | 0 |
| 3 | ONHM4222 | F | 54.7 | 22.9 | 16.0 | 10.5 | 5.7 | 7.3 | 9.0 | 4.4 | 1.8 | 8.2 | 6.6 | 10.6 | 10.4 | 14 | 9 | 3 | 14 | 10 | 0 |
| 3 | BMNH2008.988 | F | 44.3 | 17.5 | 12.7 | 9.1 | 5.4 | 5.2 | 7.3 | 3.2 | 1.3 | 6.9 | 6.1 | 8.4 | 9.6 | 12 | 9 | 3 | 14 | 11 | 0 |
| 3 | IBECN10422 | F | 53.2 | 20.4 | 15.6 | 11.1 | 6.8 | 7.1 | 9.3 | 3.9 | 2.2 | 7.6 | 7.0 | 11.3 | 11.9 | 14 | 8 | 3 | 12 | 10 | 0 |
|  | **Summary Statistics (Total)** |  |  |  |  |  |  |  |  |  |  |  |  |  |  |  |  |  |  |  |  |
|  | Mean |  | 53.8 | 22.2 | 15.8 | 10.8 | 6.2 | 6.8 | 8.9 | 4.0 | 1.9 | 8.4 | 7.3 | 10.4 | 11.7 | 13.9 | 8.2 | 2.9 | 13.3 | 10 | - |
|  | Standart Deviation |  | 3.3 | 2.2 | 1.0 | 0.7 | 0.7 | 0.6 | 0.5 | 0.3 | 0.3 | 0.6 | 0.5 | 0.8 | 1.0 | 1.3 | 0.6 | 0.3 | 0.8 | 0.6 | - |
|  | Minimum |  | 44.3 | 17.5 | 12.7 | 9.1 | 4.8 | 5.2 | 7.3 | 3.2 | 1.3 | 6.9 | 6.1 | 8.4 | 9.6 | 12 | 7 | 2 | 12 | 9 | - |
|  | Maximum |  | 58.6 | 26.1 | 17.1 | 11.8 | 7.6 | 7.7 | 9.5 | 4.4 | 2.3 | 9.3 | 8.2 | 11.3 | 13.1 | 16 | 9 | 3 | 14 | 11 | - |
