# Supplementary material for: Microendemicity in the northern Hajar Mountains of Oman and the United Arab Emirates with the description of two new species of geckos of the genus Asaccus (Squamata: Phyllodactylidae)
Source: PeerJ. 2016 Aug 18;4:e2371. doi: 10.7717/peerj.2371 (PMC4994081; doi:10.7717/peerj.2371)
Supplement: Table S3 — The asterisk (*) signalling the component indicates that is significantly different between species and its loadings in bold indicates the most important variables. Abbreviations of each morphological variable as in Table S2 and in the material and methods. [file peerj-04-2371-s003.docx]

| Trait | PC1* | PC2 | PC3 | PC4 | PC5 | PC6 |
| --- | --- | --- | --- | --- | --- | --- |
| TrL | -0.404 | -0.174 | -0.319 | 0.746 | 0.083 | 0.230 |
| HL | 0.529 | 0.252 | 0.405 | -0.162 | -0.184 | 0.514 |
| HW | -0.422 | 0.784 | -0.114 | 0.014 | -0.180 | 0.185 |
| HH | 0.131 | 0.744 | -0.247 | -0.093 | -0.025 | -0.423 |
| SL | -0.118 | -0.248 | 0.858 | 0.105 | 0.039 | -0.291 |
| SW | -0.270 | 0.499 | 0.608 | 0.383 | -0.158 | -0.006 |
| ED | 0.735 | -0.270 | -0.025 | -0.095 | -0.394 | 0.157 |
| EVD | 0.523 | 0.286 | 0.182 | -0.062 | 0.717 | 0.193 |
| LUn | **0.832** | 0.116 | -0.022 | 0.326 | -0.143 | -0.145 |
| LHu | **0.854** | -0.066 | 0.019 | 0.176 | 0.085 | -0.081 |
| LTb | **0.884** | -0.022 | -0.050 | 0.093 | -0.102 | -0.151 |
| LFe | **0.800** | 0.203 | -0.154 | 0.150 | 0.088 | 0.032 |
| Eigenvalue | 4.381 | 1.787 | 1.506 | 0.931 | 0.814 | 0.728 |
| Variability (%) | 36.511 | 14.894 | 12.554 | 7.758 | 6.784 | 6.069 |
| Cumulative (%) | 36.511 | 51.405 | 63.959 | 71.717 | 78.502 | 84.570 |
