# Supplementary material for: Microendemicity in the northern Hajar Mountains of Oman and the United Arab Emirates with the description of two new species of geckos of the genus Asaccus (Squamata: Phyllodactylidae)
Source: PeerJ. 2016 Aug 18;4:e2371. doi: 10.7717/peerj.2371 (PMC4994081; doi:10.7717/peerj.2371)
Supplement: Table S4 — (A) A. margaritae sp. nov. versus A. gardneri sp. nov.; (B) A. margaritae sp. nov. versus A. caudivolvulus and (C) A. gardneri sp. nov. versus A. caudivolvulus. P-values in bold indicate high level of statistical significance. Abbreviations of each morphological variable as in Table S2 and in the material and methods. [file peerj-04-2371-s004.docx]

|  | **TrL** | **HL** | **HW** | **HH** | **SL** | **SW** | **ED** | **EVD** | **LUn** | **LHu** | **LTb** | **LFe** | **Trow** | **LT4** | **ST4** | **ULS** | **LLS** |
| --- | --- | --- | --- | --- | --- | --- | --- | --- | --- | --- | --- | --- | --- | --- | --- | --- | --- |
| R² | 0.055 | 0.154 | 0.083 | 0.009 | 0.003 | 0.029 | 0.288 | 0.090 | 0.421 | 0.306 | 0.431 | 0.301 | 0.004 | 0.477 | 0.071 | 0.045 | 0.017 |
| F | 3.153 | 9.823 | 4.860 | 0.496 | 0.164 | 1.620 | 21.857 | 5.323 | 39.187 | 23.841 | 40.931 | 23.204 | 0.221 | 49.264 | 4.150 | 2.564 | 0.920 |
| Pr > F | 0.081 | **0.003** | 0.032 | 0.484 | 0.687 | 0.209 | **< 0.0001** | 0.025 | **< 0.0001** | **< 0.0001** | **< 0.0001** | **< 0.0001** | 0.640 | **< 0.0001** | 0.047 | 0.115 | 0.342 |

**A)**

|  | **TrL** | **HL** | **HW** | **HH** | **SL** | **SW** | **ED** | **EVD** | **LUn** | **LHu** | **LTb** | **LFe** | **Trow** | **LT4** | **ST4** | **ULS** | **LLS** |
| --- | --- | --- | --- | --- | --- | --- | --- | --- | --- | --- | --- | --- | --- | --- | --- | --- | --- |
| R² | 0.035 | 0.128 | 0.065 | 0.004 | 0.003 | 0.027 | 0.273 | 0.080 | 0.412 | 0.411 | 0.446 | 0.203 | 0.276 | 0.253 | 0.138 | 0.030 | 0.016 |
| F | 0.584 | 2.342 | 1.105 | 0.063 | 0.046 | 0.452 | 6.019 | 1.397 | 11.218 | 11.171 | 12.896 | 4.079 | 6.094 | 5.420 | 2.571 | 0.495 | 0.263 |
| Pr > F | 0.456 | 0.145 | 0.309 | 0.805 | 0.834 | 0.511 | 0.026 | 0.254 | **0.004** | **0.004** | **0.002** | 0.061 | 0.025 | 0.033 | 0.128 | 0.492 | 0.615 |

**B)**

|  | **TrL** | **HL** | **HW** | **HH** | **SL** | **SW** | **ED** | **EVD** | **LUn** | **LHu** | **LTb** | **LFe** | **Trow** | **LT4** | **ST4** | **ULS** | **LLS** |
| --- | --- | --- | --- | --- | --- | --- | --- | --- | --- | --- | --- | --- | --- | --- | --- | --- | --- |
| R² | 0.002 | 0.005 | 0.003 | 0.000 | 0.000 | 0.001 | 0.012 | 0.003 | 0.021 | 0.012 | 0.021 | 0.014 | 0.176 | 0.088 | 0.003 | 0.003 | 0.025 |
| F | 0.095 | 0.244 | 0.123 | 0.019 | 0.003 | 0.033 | 0.562 | 0.130 | 0.965 | 0.566 | 0.996 | 0.657 | 9.802 | 4.420 | 0.126 | 0.141 | 1.187 |
| Pr > F | 0.760 | 0.624 | 0.727 | 0.891 | 0.954 | 0.858 | 0.457 | 0.720 | 0.331 | 0.456 | 0.324 | 0.422 | **0.003** | 0.041 | 0.724 | 0.709 | 0.282 |

**C)**
