# Supplementary figures and images for: Microendemicity in the northern Hajar Mountains of Oman and the United Arab Emirates with the description of two new species of geckos of the genus Asaccus (Squamata: Phyllodactylidae)
Source: PeerJ. 2016 Aug 18;4:e2371. doi: 10.7717/peerj.2371 (PMC4994081; doi:10.7717/peerj.2371)

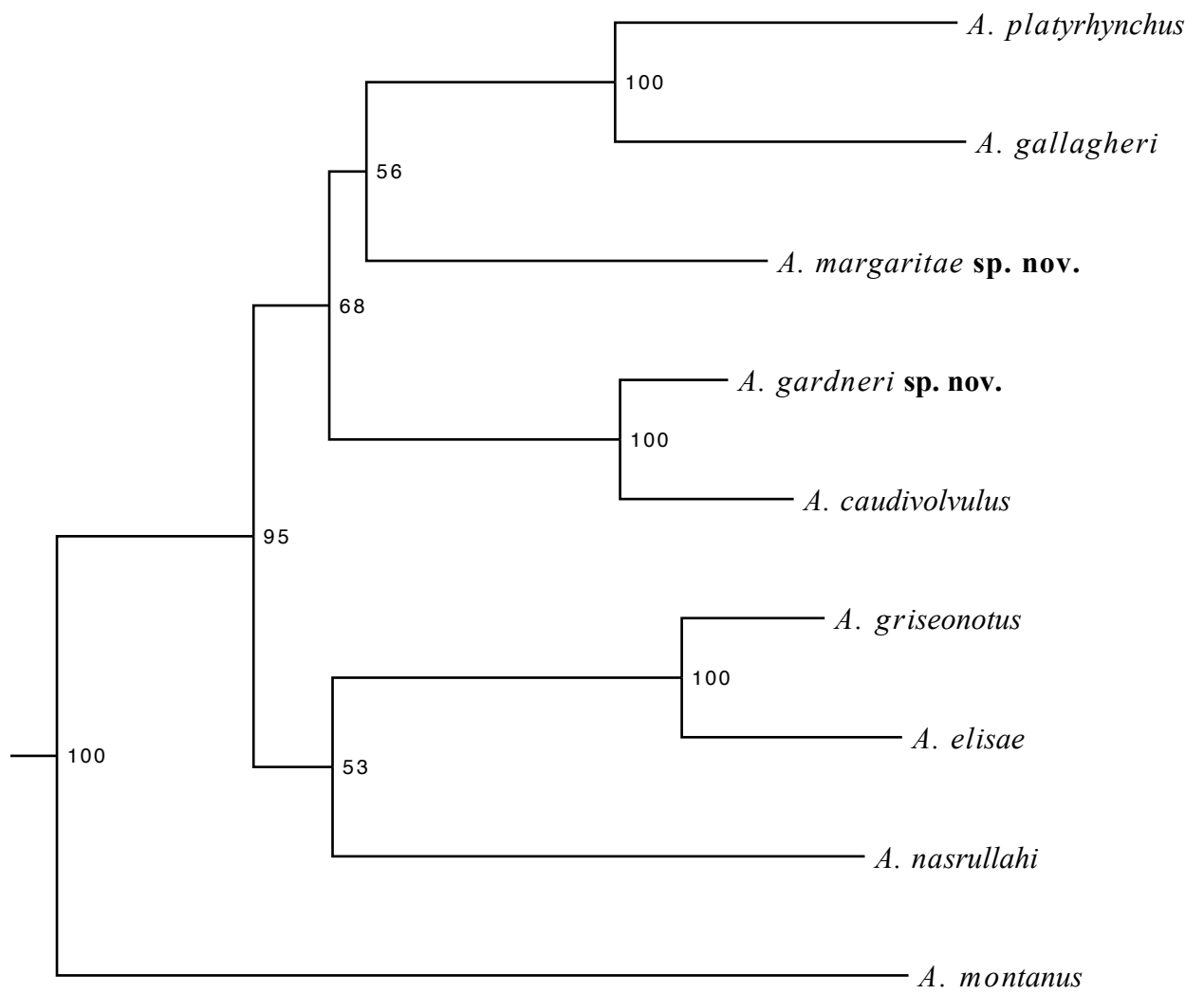

0.04

Supplement: Figure S1 — The phylogeny is based on the concatenated sequences of two mitochondrial (12S and cytb) and three nuclear (c-mos, MC1R and ACM4) genes. Bootstrap values ≥70% of the ML analysis are shown next to the nodes. [file peerj-04-2371-s005.pdf]

A) *c-mos*

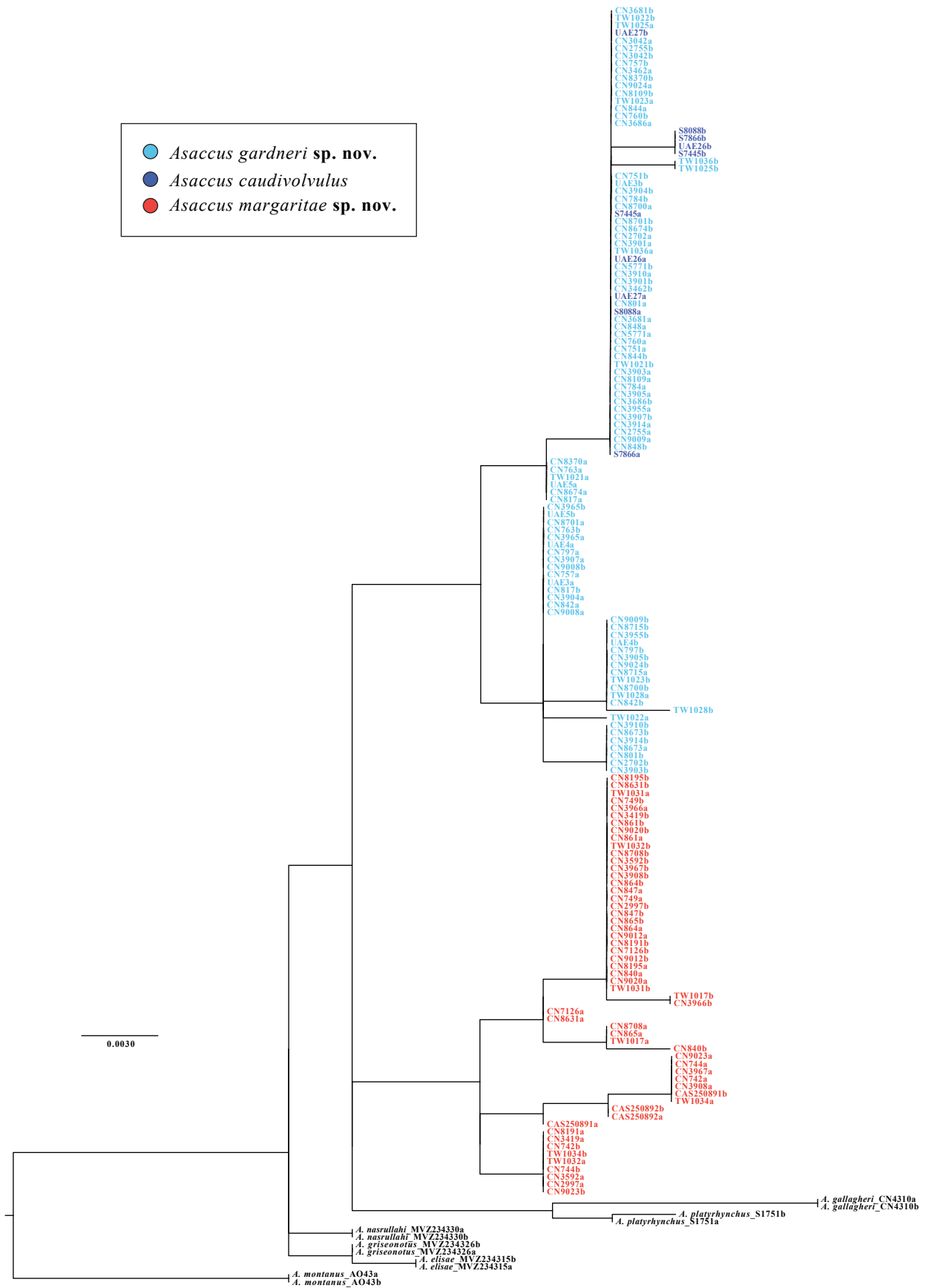

B) MCIR

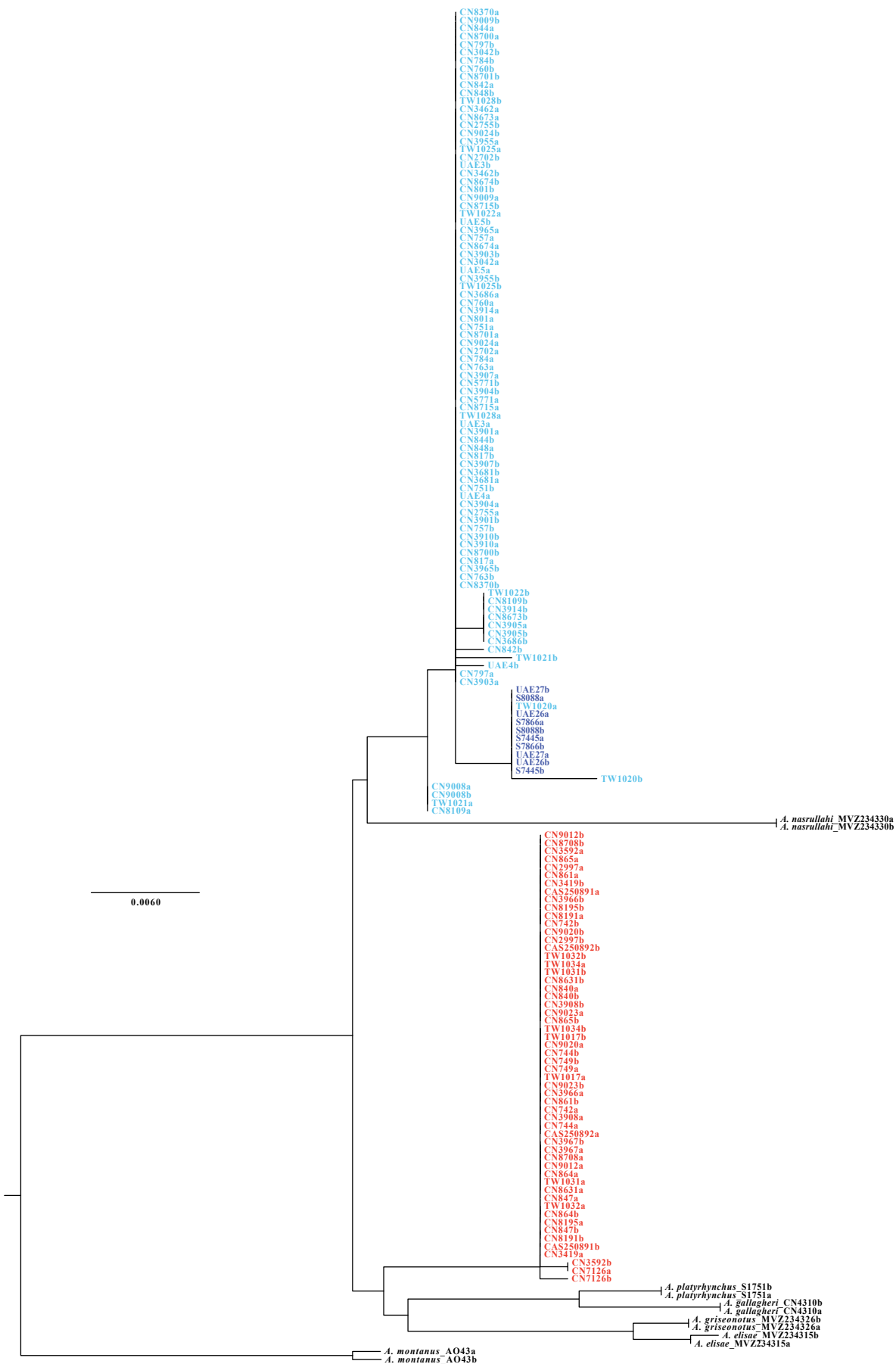

C) *ACM4*

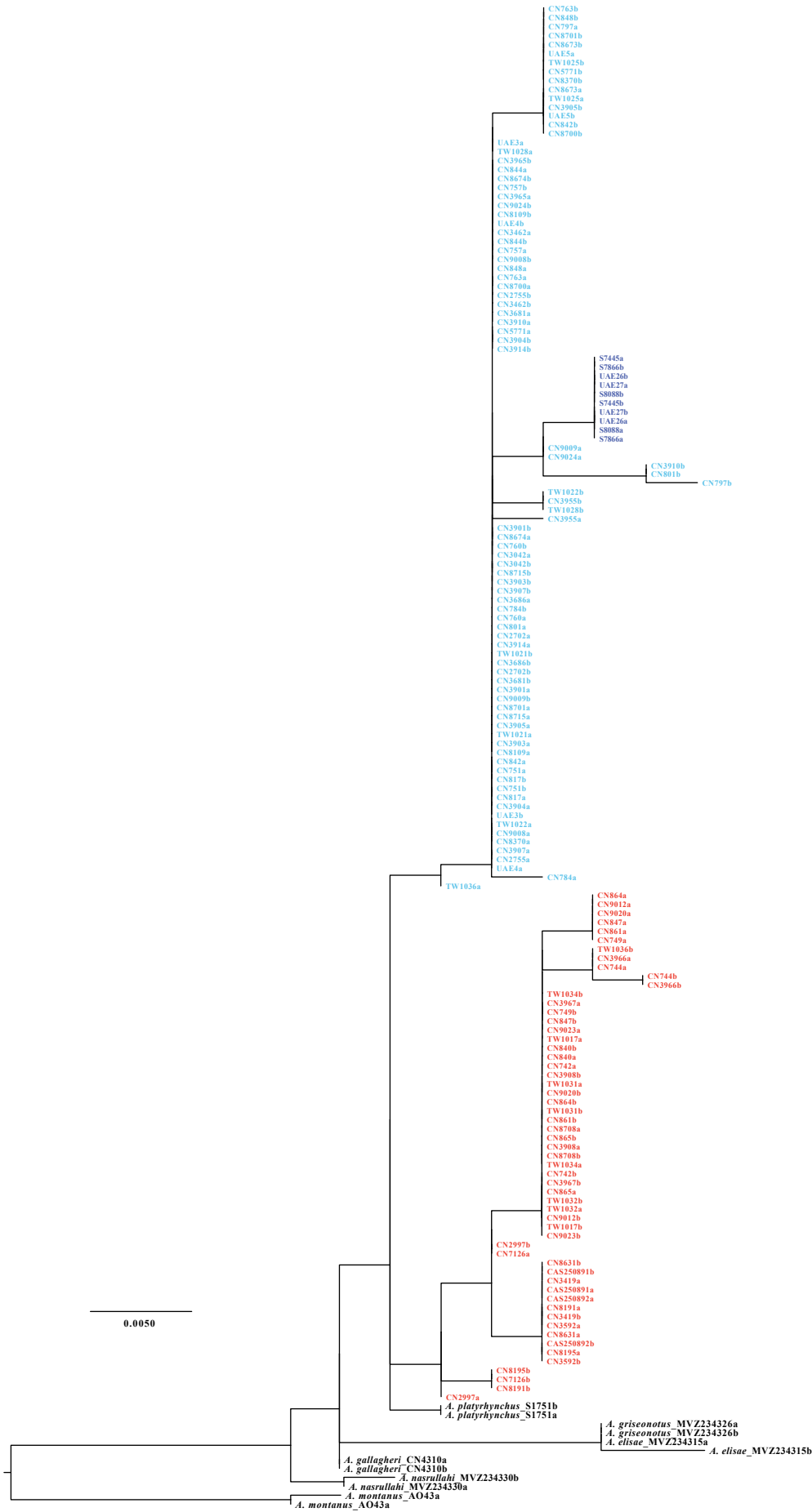

Supplement: Figure S2 — (A) c-mos, (B) MC1R, (C) ACM4. The dataset used were phased in order to show the two alleles of each specimen. All the haplotypes of the three nuclear genes are private for all Asaccus species, with the only exception of Asaccus caudivolvulus and Asaccus gardneri sp. nov., which share one haplotype in the CMOS gene and one haplotype in the MC1R gene (see Fig. 2). [file peerj-04-2371-s006.pdf]
